# Adiponectin protects obesity-related glomerulopathy by inhibiting ROS/NF-κB/NLRP3 inflammation pathway

**DOI:** 10.1186/s12882-021-02391-1

**Published:** 2021-06-10

**Authors:** Xiaohong Xu, Xiaolin Huang, Liexiang Zhang, Xiaoli Huang, Zihan Qin, Fei Hua

**Affiliations:** 1 Department of Endocrinology, The Third Affiliated Hospital of Soochow University, No.185 Bureau Front Street, 213003 Changzhou City, China; 2 Department of Nephrology, The Affiliated Suqian Hospital of Xuzhou Medical University, Suqian City, China; 3 Department of Nephrology, Suqian People’s Hospital, Nanjing Drum Tower Hospital Group, Suqian City, China; 4 Department of Neurosurgery, The Affiliated Suqian Hospital of Xuzhou Medical University, Suqian City, China; 5 Department of Neurosurgery, Suqian People’s Hospital, Nanjing Drum Tower Hospital Group, Suqian City, China

**Keywords:** Adiponectin, Obesity-related glomerulopathy, ROS, NF-κB, NLRP3 inflammation

## Abstract

**Background:**

Adiponectin is an adipocytokine that plays a key regulatory role in glucose and lipid metabolism in obesity. The prevalence of obesity has led to an increase in the incidence of obesity-related glomerulopathy (ORG). This study aimed to identify the protective role of adiponectin in ORG.

**Methods:**

Small-interfering RNA (siRNA) against the gene encoding adiponectin was transfected into podocytes. The oxidative stress level was determined using a fluorometric assay. Apoptosis was analyzed by flow cytometry. The expressions of podocyte markers and pyrin domain containing protein 3 (NLRP3) inflammasome-related proteins were measured by qRT-PCR, immunohistochemistry, and Western blot.

**Results:**

Podocytes treated with palmitic acid (PA) showed downregulated expressions of podocyte markers, increased apoptosis, upregulated levels of NLRP3 inflammasome-related proteins, increased production of inflammatory cytokines (IL-18 and IL-1β), and induced activation of NF-κB as compared to the vehicle-treated controls. Decreased adiponectin expression was observed in the serum samples from high fat diet (HFD)-fed mice. Decreased podocin expression and upregulated NLRP3 expression were observed in the kidney samples from high fat diet (HFD)-fed mice. Treatment with adiponectin or the NLRP3 inflammasome inhibitor, MCC950, protected cultured podocytes against podocyte apoptosis and inflammation. Treatment with adiponectin protected mouse kidney tissues against decreased podocin expression and upregulated NLRP3 expression. The knockout of adiponectin gene by siRNA increased ROS production, resulting in the activation of NLRP3 inflammasome and the phosphorylation of NF-κB in podocytes. Pyrrolidine dithiocarbamate, an NF-κB inhibitor, prevented adiponectin from ameliorating FFA-induced podocyte injury and NLRP3 activation.

**Conclusions:**

Our study showed that adiponectin ameliorated PA-induced podocyte injury *in vitro* and HFD-induced injury *in vivo* via inhibiting the ROS/NF-κB/NLRP3 pathway. These data suggest the potential use of adiponectin for the prevention and treatment of ORG.

**Supplementary Information:**

The online version contains supplementary material available at 10.1186/s12882-021-02391-1.

## Background

Obesity is a leading public health problem with increasing global incidence rates. According to the Chinese Chronic Disease Surveillance (2010), the prevalence of abdominal obesity and obesity in Chinese adults was 40.7 and 12.0 %, respectively [1]. Obesity is a dynamic process, during which the amount and the distribution of adiposity undergo significant change. Different adipose tissue depots play different roles in the development of other diseases, such as cardiovascular disease and renal disease. Morbid obesity has been shown to adversely affect cardiac and renal function [2]. Obesity-related glomerulopathy (ORG) is a well-characterized renal complication of obesity, referring to proteinuria and glomerulomegaly in obese patients who show no clinical and histopathological evidence of other renal diseases [3].

The incidence rate of ORG is correlated with the prevalence of obesity [4, 5]. Chen et al. analyzed 10,093 renal biopsies collected from 2002 to 2006 and found that the incidence of ORG increased from 0.62 to 1.00 % in 5 years [6]. The pathogenesis of obesity is associated with adipose tissue dysfunction, such as increased inflammation level, instead of changes in adipose tissue volume; therefore, BMI may not be a reliable marker for metabolic syndrome [7]. Current evidence suggests that the pathogenic mechanisms of ORG involve renal hemodynamic changes, insulin resistance (IR), activation of the renin-angiotensin-aldosterone system (RAAS), sympathetic nervous system stimulation, and oxidative stress. RAAS hyperactivation leads to an increase in the production of angiotensin II (Ang II), which regulates water and sodium balance and blood pressure by binding to Ang II type 1 receptor [8]. The long-term overexpression of Ang II increases renal perfusion, induces inflammation, aggravates renal pathological changes, and ultimately leads to proteinuria progression. Compared to healthy subjects, the renal tissues of ORG patients showed higher expressions of tumor necrosis factor-α, interleukin (IL)-1, and IL-6, which resulted in glomerular sclerosis and the impairment of renal structure and function [9].

Podocytes are differentiated visceral epithelial cells with minimal regenerative capacity [10]. The number and integrity of podocytes are critical for the maintenance of normal glomerular filtration [11]. Stress-induced dysfunction, injury, and apoptosis of podocytes are critical for the pathogenesis of proteinuria and chronic nephropathy [12, 13]. Therefore, it is vital to protect podocytes from damage and apoptotic cell death.

Adiponectin is an adipocytokine mainly secreted from adipose tissues with anti-atherosclerotic, anti-inflammatory, and anti-diabetic properties [10]. It plays essential regulatory roles in glucose and lipid metabolism, and is inversely correlated with obesity. Prior studies have shown that adiponectin undergoes a substantial increase after weight loss [14] and *vice versa*. There are three adiponectin receptors (AdipoR): AdipoR1, AdipoR2, and T-cadherin. The first two receptors share a similar structure, with seven transmembrane domains and an intracellular zinc binding motif capable of regulating downstream cellular signaling [15]. AdipoR1 is widely expressed in human cells with the most abundant expression observed in skeletal muscles. AdipoR2 is mainly present in the liver. Globular adiponectin shows the highest affinity to AdipoR1; therefore, in animal studies, it acts mainly in muscle cells [16]. T-cadherin, lacking a transmembrane structure, is considered a binding protein for the high molecular weight form. Previous studies have shown that T-cadherin is a major binding partner for adiponectin, causing its accumulation in the heart, vascular endothelium, and skeletal muscles. The direct downstream effect of this binding is still under investigation [15, 17, 18].

AdiopoR1 has been shown to increase the expression of 5’adenosine monophosphate-activated protein kinase, while AdipoR2 activates the peroxisome proliferator-activated receptor alpha pathway [19]. Compared to wild-types, adiponectin-knockout mice subjected to subtotal nephrectomy exhibited significantly worsened tubulointerstitial fibrosis, glomerular hypertrophy, and urine albumin excretion [20], whereas the infusion of adiponectin in mice effectively reduced oxidative stress in kidney tissues and reversed albuminuria [19].

The activation of the recombinant NLR family, pyrin domain containing protein 3 (NLRP3) inflammasome by damage-associated molecular patterns (DAMPs), such as dietary free fatty acids (FFAs), is a key event in obesity-induced inflammation [21, 22]. Palmitic acid (PA) is the most common dietary saturated fat and makes up of approximately 20 % of the total serum FFAs. It acts as a first or second signal for the activation of inflammatory pathways [23]. However, the mechanisms underlying the involvement of FFAs in inflammation have not been fully understood.

This study aims to investigate the protective role of adiponectin in obesity-induced ORG using a cell model of FFA-induced cell injury and a mouse model of obesity.

## Methods

### Preparation of bovine serum albumin (BSA)-conjugated palmitic acid

Palmitic acid was first dissolved in pure water to a final concentration of 5 mM with robust shaking at 70 °C for at least 30 min. Once dissolved, the solution was mixed with a filtered 5 % fatty acid- and endotoxin-free BSA solution in phosphate buffer saline (PBS) pre-heated at 70 °C to a final concentration of 2.5 mM. Aliquots of the 2.5 mM stock solution were kept at -20 °C for further use.

### Cell culture and treatment

The immortalized murine podocyte clone 5 (MPC5) cell line (Shanghai Haling Biotechnology) were maintained in Dulbecco’s Modified Eagle Medium (DMEM) containing 10 % fetal bovine serum (FBS), 4.5 g/l glucose, and 1 % streptomycin/penicillin in a 37 °C/5 % CO_2_ incubator. The low-serum medium (1 % FBS) was used for transfection experiments and assays performed in MPC5 cells. Treatment with PA at high levels has been commonly used to establish cell models of FFA-induced inflammation [24]. The control group was treated with identical concentrations of BSA. MPC5 cells were treated with vehicle or recombinant mouse adiponectin (2 µg/ml, R&D, #5095-AC-050) for 24 h in the absence or presence of sodium palmitate (Absin, #abs42027533). All experiments were repeated three times, each performed in six replicates.

Small-interfering RNAs (siRNAs) against adiponectin and a negative control (NC) sequence were obtained from GenePharma (Shanghai, China). The sequences of siRNAs were: adiponectin siRNA (5′–3′): sense: CUGUUCCCAAUGUACCCAUTT, antisense: AUGGGUACAUUGGGAACAGTT; NC siRNA (5′–3′): sense: UUCUCCGAACGUGUCACGUTT, antisense: ACGUGACACGUUCGGAGAATT. MPC5 cells were transfected with siRNAs against adiponectin (100 nM) for 48 h using the Lipofectamine RNAiMAX Transfection Reagent (Invitrogen, CA, USA), incubated with an NLRP3 inhibitor MCC950 (100 nM, R&D, #5479/10), or treated with an NF-κB inhibitor, pyrrolidine dithiocarbamate (PDTC, 50 µM, Sigma, #P8765) for 24 h followed by sodium palmitate treatment. The transfection efficiency was evaluated by Western blot and quantitative real-time PCR (qRT-PCR). The siRNA with the most robust inhibitory effect (adipoQ-mus-492) was used for subsequent experiments.

### Animal model

Animal experiments were approved by the Ethics Committee of Animal Experiments of Soochow University. All experiments were performed according to the NIH guidelines for the care and use of laboratory animals. This study was carried out in compliance with the ARRIVE guidelines. Six-week old male C57BL/6J mice (Sippe-Bk Lab Animal Co., Shanghai, China) were maintained in a specific pathogen-free laboratory animal facility with a 12-h light-dark cycle. They were housed in individual metabolic cages with *ad libitum* access to water and food through 12 weeks of age. Then mice were randomly divided into 4 groups (N = 6 per group), namely, control (CON) group, CON + gAd group, HFD group, and HFD + gAd group. The mouse obesity model was established by feeding animals with HFD (Research Diets, #D12492, New Brunswick, USA) containing (kcal) 20 % carbohydrate, 60 % fat, and 20 % protein for six weeks. The CON and CON + gAd groups were fed with a regular chow diet (Research Diets, #D12450B) containing (kcal) 70 % carbohydrate, 10 % fat, and 20 % protein. Mice at six-week-old were injected with adiponectin or an equal amount of normal saline (10 µg/kg/d) via tail vein injection. After six weeks, mice were anesthetized using 3 % isoflurane and euthanized with an overdose injection of pentobarbital. The serum samples were collected and the kidneys were dissected. At 6, 8, 10, and 12 weeks of age, the body weight of each animal was measured and the levels of fasting blood glucose, serum creatinine, and blood lipids (total cholesterol, low-density lipoprotein, high-density lipoprotein, triglyceride) were detected using commercially available assay kits (NJJCBIO, China). The level of serum adiponectin level was assessed at 6 and 12 weeks of age using commercially available assay kits (NJJCBIO, China). The 24-h urine samples were collected every 2 weeks. The urinary albumin content was determined using an mALB Assay Kit (NJJCBIO, #E038-1-1). A creatinine assay kit (NJJCBIO, #C011-2-1) was used to assess urinary creatinine concentration. The urinary albumin excretion was shown as the ratio of urinary albumin concentration to creatinine concentration (ACR, µg/mg). The paraffin-embedded renal cortices Sec. (3 μm) were incubated with 3 % H_2_O_2_ for 10 min. Images of HE staining were captured using a Zeiss microscope (400× magnification) equipped with a Spot Insight camera. Immunofluorescence staining was used to detect the expressions of podocin and NLRP3 in renal cortices sections.

### Cell proliferation assay

The proliferation of podocytes was determined using a Cell Counting Kit-8 (CCK-8) assay (Beyotime, #C0038, Shanghai, China). Cells (1–5 × 10^5^ cells/ml) were cultured in 96-well plates under the culture condition described above. After 24 or 48 h, podocytes were incubated with a mixture of CCK-8 solution and serum-free DMEM (1:10) at 37 °C for 1 h in a 5 % CO_2_ atmosphere. The absorbance value was detected at 450nm by a microplate reader (Thermo Scientific, Waltham, USA).

### Boron-dipyrromethene (BODIPY) lipid probes and Oil Red O staining

The lipid intake was measured by BODIPY lipid probes and Oil Red O staining. To measure the fatty acid intake by BODIPY probes, podocytes were incubated with 10 µg/ml BODIPY lipid probes (BODIPY500/510 C1, C12, Invitrogen) at 37 °C for 1 h. After washing three times with PBS, cells were examined under a fluorescence microscope. In preparation for Oil Red O staining, podocytes were fixed in 4 % formaldehyde for 15 min and then stained with Oil Red O working solution for half an hour. Then cells were washed with 60 % isopropanol for 5 s followed by the treatment with hematoxylin for 5 min.

### ELISA

The levels of IL-18 and IL-1β in cell culture supernatants were determined using ELISA kits (Abcam #ab216165, and R&D #VAL601). The concentrations of IL-18 and IL-1β in mouse kidney samples were measured using Mouse IL-18 Valukine ELISA Kit (Abcam, #ab216165) and Mouse IL-1β Valukine ELISA Kit (R&D, #VAL601), respectively, following the manufacturer’s protocols.

### Reactive oxygen species (ROS) assay

The generation of ROS in podocytes was assessed using fluorometry assay via intracellular oxidation of dichlorodihydrofluorescein diacetate (Beyotime, #s0033). Cells (1 × 10^6^/ml) were cultured in a 6-well plate for 24 h. Following a 30-min incubation with dichlorodihydrofluorescein diacetate (50 µg/ml) at 37 °C, cells were harvested for flow cytometry analysis (Beckman Coulter, CA, USA). The fluorescent product 2′,7′-dichlorofluorescein was detected at a wavelength of 480 nm. The results were analyzed using BD CellQuest software (Becton Dickinson, San Jose, USA).

### Western blot

MPC5 cells were lysed in RIPA buffer containing 1 mM phenylmethylsulfonyl fluoride (Beyotime). The BCA Protein Assay Kit (Beyotime) was used to measure the protein concentration in each sample. Equal amounts of protein samples were loaded, separated by 10 or 12 % SDS-PAGE, and subsequently electrophoretically transferred to polyvinylidene fluoride membranes (Millipore, Stafford, USA). After blocking with Tris-buffered saline containing 5 % dry milk and 0.1 % Tween20 for 1 h, the membranes were incubated with the following primary antibodies overnight at 4 °C: nephrin (Abcam, #ab216341), podocin (Abcam, #ab50339), desmin (Proteintech, #60226-1-Ig), nuclear factor-κB (NF-κB)/p65 (CST, #8242T), p-NF-κB/p-p65 (CST, #3033T), adapter protein apoptosis-associated speck-like protein (ASC, CST, #67824T), NLRP3 (Proteintech, #19771-1-AP), caspase-1 (Proteintech, #22915-1-AP), adiponectin (Proteintech, #21613-1-AP). All antibodies were diluted at 1:1000. After washing with TBS, the blots were incubated with a horseradish peroxidase-conjugated secondary antibody (goat anti-rabbit IgG H&L or goat anti-mouse IgG H&L, Beyotime, #A0208 and #A0216). The anti-β-actin antibody (1:1000, Cell Signaling Technology, #4970) was used as the loading control. The bands were visualized using an Enhanced Chemiluminescence Kit (Thermo Fisher) and a ChemiDoc XRS Plus Luminescent Image Analyzer (Bio-Rad). The band intensities were determined using Image J software.

### qRT-PCR

Total RNAs were extracted from MPC5 cells by Trizol reagent (Invitrogen). Cells were added with Trizol reagent. After 5–10 min, the mixture was transferred to an EP tube and treated with 1/5 volume of chloroform. After 10–15 min incubation at 4 ℃, the sample was centrifuged at 4℃ for 15 min. Then the supernatant was removed and added to an equal volume of isopropanol. After 10-min incubation at 4 ℃, the sample was centrifuged at 12,000 rpm for 10 min. Then the supernatant was discarded. The precipitate was added with pre-cooled 75 % alcohol and then centrifuged at 4 ℃ for 5 min. Subsequently, the supernatant was discarded. After instant centrifugation, the residual liquid was discarded. 20 µL DEPC water was added to the sample to dissolve the precipitate. The RNA concentration was measured. The complementary DNAs were synthesized using an iScriptTM cDNA Synthesis Kit (Bio-Rad, Hercules, USA). The qRT-PCR was performed using SYBR Green Master Mix (Bio-Rad) and the ABI 7900HT Real-Time PCR Detection System (Applied Biosystems, CA, USA). Relative mRNA expression was analyzed using the 2^−ΔΔCt^ method and normalized to β-actin expression.The primer sequences are shown in Table 1.

**Table 1:** The sequences of primers for qRT-PCR

	Species	Forward	Reverse
**nephrin**	Mouse	TAGTGGACGTGGACGAGGTT	GAGGACAAGAAGCCACTCGC
**podocin**	Mouse	GGATGGCGGCTGAGATTCTG	AAACCACAGTGGCTGGCTTC
**desmin**	Mouse	CCAAGCAGGAGATGATGGAATA	CATCCTTTAGGTGTCGGATCTC
**adiponectin**	Mouse	CCAATGTACCCATTCGCTTTAC	GAAGTAGTAGAGTCCCGGAATG
**NLRP3**	Mouse	GTGGTGACCCTCTGTGAGGT	TCTTCCTGGAGCGCTTCTAA
**caspase-1**	Mouse	TGACAAGAAGGCAAAGGCCG	ACCTCGTCCACGTCCACTAC
**NF-κB**	Mouse	TCGAGTCTCCATGCAGCTACGG	CGGTGGCGATCATCTGTGTCTG
**β-actin**	Mouse	CATCCGTAAAGACCTCTATGCCAAC	ATGGAGCCACCGATCCACA

### Flow cytometry

Apoptosis was analyzed by Annexin V-FITC/PI Apoptosis Kit (Bioworld, Nanjing, China). In brief, podocytes were digested with trypsin, washed three times with PBS, and treated with 100 µL binding buffer. After incubating with propidium iodide (5 µL) and Annexin V-FITC (5 µL) for half an hour in the dark, cells were analyzed by flow cytometry (Beckman Coulter). The percentage of apoptotic cells was determined by the Annexin V/PI ratio. The Annexin V staining intensity was set as the horizontal axis and the PI staining intensity was set as the vertical axis. The survival, apoptosis, and necrosis of MPC5 cells in different groups were compared.

### Immunofluorescence

The paraffin-embedded renal cortices Sec. (3 μm) were incubated with primary antibodies against podocin (NPHS2, Abcam, # ab50339, 1:200) and NLRP3 (NALP3, BIOSS, #bs-6655R, 1:200) overnight at 4 °C. Ten fields of view (400 × magnification) per slide were randomly selected for analysis and the percentage of positively-stained area over the whole glomerular area was calculated using Image J Software (National Institutes of Health).

### Statistical analysis

All data were analyzed using Prism software version 7.0 (GraphPad, San Diego, USA) and expressed as means ± SEM. The normality of the data was evaluated using the Shapiro-Wilk test. The statistical analysis among different groups were performed using either an unpaired Student t-test or a one-way analysis of variance with Tukey’s post hoc test. A *p* ≤ 0.05 was considered statistically significant. The symbols in the figures indicate the significance level.

## Results

### PA reduced cell viability, increased lipid accumulation, and induced cell apoptosis in podocytes

**Fig. 1:**
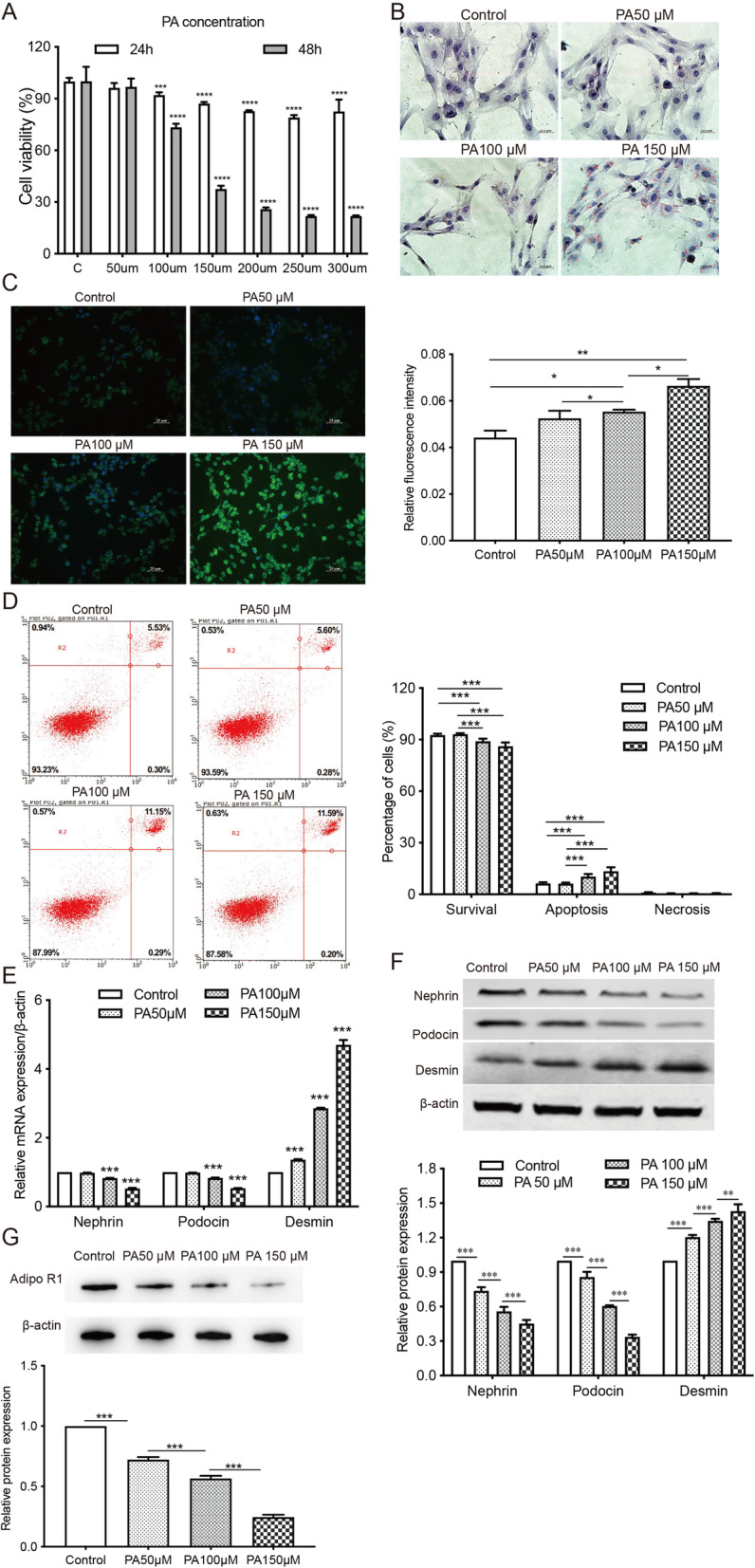
PA reduces cell viability, induces apoptosis, and promotes podocyte injury in MPC5 cells. MPC5 cells were exposed to different concentrations of PA (50, 100, 150, 200, 250, and 300 µM) for 24 or 48 h. **a**, CCK-8 assay was performed to evaluate the impact of PA on cell viability. *** *p* < 0.001, **** *p* < 0.0001 vs. the control group. **b**-**c**, Oil Red O staining and BODIPY lipid probes were used to assess intracellular lipid accumulation in cells treated with PA at 50, 100, and 150µM. * *p* < 0.05, ** *p* < 0.01 vs. the control group. **d**, The apoptotic cells were detected by flow cytometry. *** *p* < 0.001 vs. controls. **e**, qRT-PCR analysis of podocyte markers (podocin and nephrin) and a glomerular podocyte lesion indicator (desmin) in MPC5 cells. *** *p* < 0.001 vs. controls. **f**, The protein expressions of nephrin, podocin, desmin in all groups of podocytes were assessed by Western Blot. ** *p* < 0.01, *** *p* < 0.001 vs. the control group. **g**, The protein expression of adipoR1 in podocytes was assessed by Western blot. *** *p* < 0.001 vs. the control group. Data are presented as mean ± SEM from three independent experiments. PA: palmitic acid

MPC5 cells were exposed to PA at 50, 100, 150, 200, 250, and 300 µM for 24 or 48 h. PA decreased the viability of MPC5 cells in a dose-dependent and time-dependent manner (Fig. 1 a). By staining podocytes with BODIPY lipid probes and Oil Red O dye, we found that the intracellular lipid deposition was gradually and significantly increased with PA treatment at increasing doses (Fig. 1b c). The results from flow cytometry demonstrated that PA dose-dependently induced apoptosis of podocytes (Fig. 1d). The mRNA and protein levels of podocyte markers, nephrin, and podocin, were decreased with increasing PA concentration (Fig. 1e f). The mRNA and protein expressions of desmin, an indicator of glomerular podocyte lesion, in podocytes were significantly elevated after the exposure to PA for 24 h (Fig. 1e f). The protein expression of adipoR1 was also downregulated with the increase in PA concentration (Fig. 1g). MPC5 cells were treated with PA at 150 µM for 24 h in the following experiments.

### PA induced NLRP3 inflammasome activation and ROS production in podocytes

**Fig. 2:**
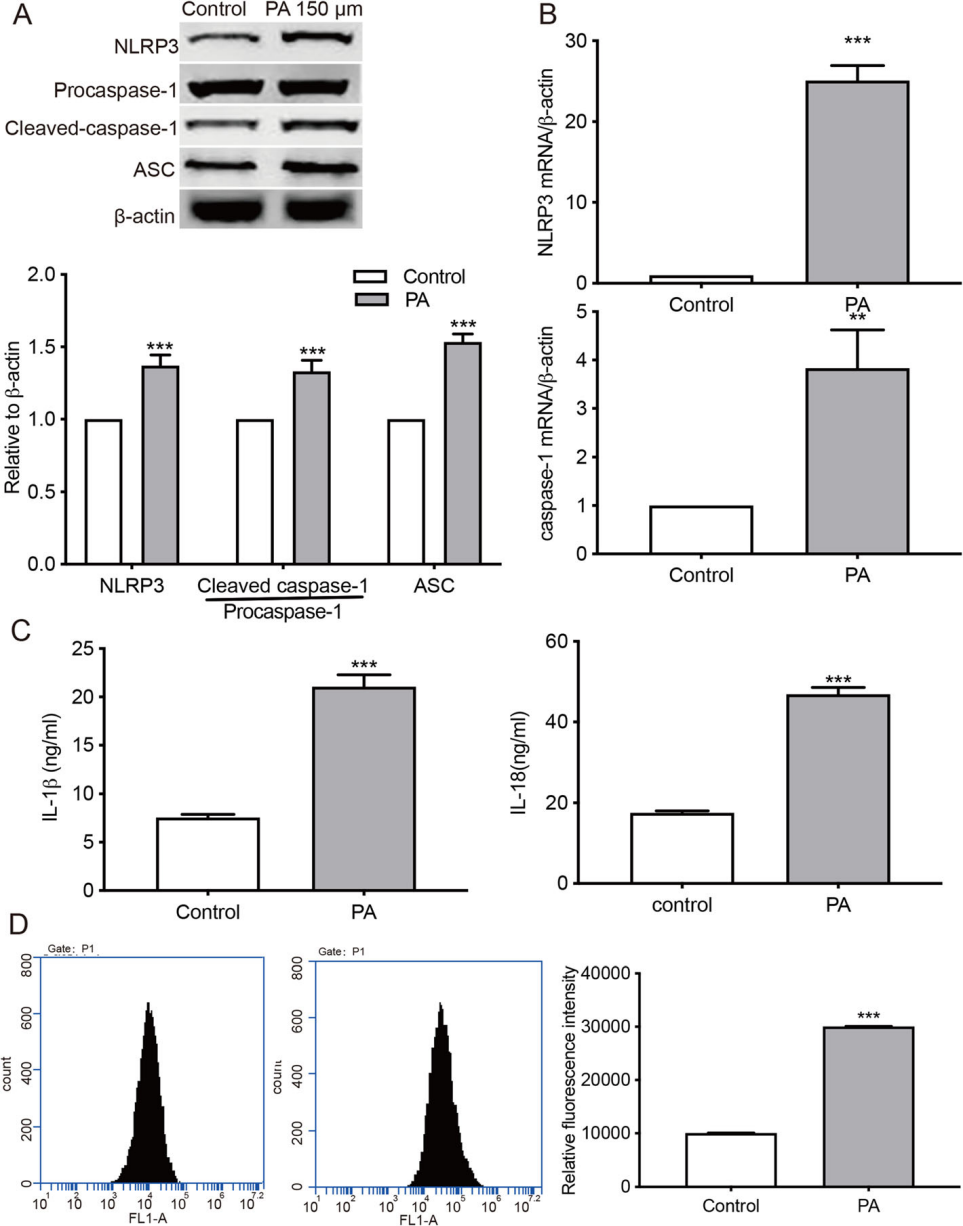
PA induces the activation of NLRP3 inflammasome and oxidative stress in MPC5 cells. Cells were treated with 150µM PA for 24 h. **a**, Western Blot analysis of NLRP3, procaspase-1, cleaved caspase-1, and ASC. *** *p* < 0.001 vs. the control group. **b**, The mRNA levels of NLRP3 inflammasome-related proteins were detected by qRT-PCR analysis. ** *p* < 0.01, *** *p* < 0.001 vs. the control group. **c**, ELISA assays were performed to measure the contents of IL-18 and IL-1β in cell culture supernatants. *** *p* < 0.001 vs. the control group. **d**. ROS production was assessed using a fluorometry assay. *** *p* < 0.001 vs. the control group. Data are presented as mean ± SEM from three independent experiments

The expression of NLRP3 inflammation-related proteins, including NLRP3, ASC, procaspase-1, and cleaved caspase-1, were analyzed by Western blot. Cells treated with 150µM PA showed significantly upregulated levels of these proteins compared with the control group (Fig. 2 a). The mRNA levels of NLRP3 and caspase-1 were also significantly higher in PA-treated group in comparison to the controls (Fig. 2b). Moreover, the secretion of IL-18 and IL-1β in PA-treated cells was significantly increased compared to the control group (Fig. 2 c). PA-treated cells also showed increased ROS production when compared to the control group (Fig. 2d).

### Adiponectin decreased NLRP3 inflammasome activation, suppressed NF-κB signaling, inhibited ROS production, and ameliorated podocyte injury in PA-induced podocytes

**Fig. 3:**
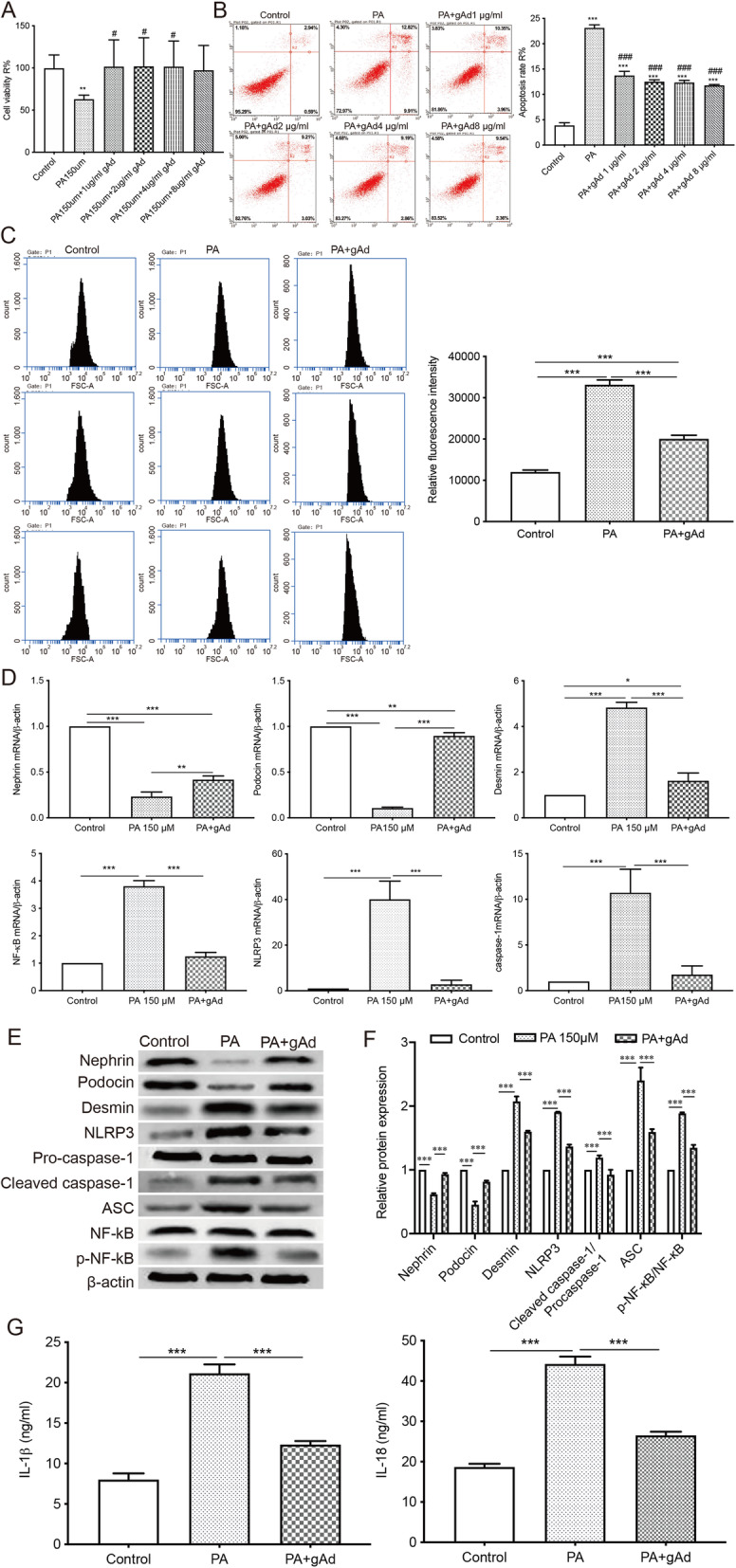
Adiponectin attenuates PA-induced apoptosis, NLRP3 inflammasome activation, and podocyte injury in vitro. **a**, The viability of cells treated with adiponectin at different concentrations (1, 2, 4, 8 µg/ml) was evaluated by CCK-8 assay. ** *p* < 0.01 vs. the control group, ^#^*p* < 0.05 vs. the PA group. **b**, The apoptosis rate was determined by flow cytometry. *** *p* < 0.001 vs. the control group, ^###^*p* < 0.001 vs. the PA group. **c**, ROS production was assessed by a fluorometric assay. *** *p* < 0.001 vs. the control group. **d**, qRT-PCR analysis of nephrin, podocin, desmin, NLRP3, caspase-1, and NF-κB. * *p* < 0.05, ** *p* < 0.01, *** *p* < 0.001 vs. controls. **e**-**f**, Western Blot was performed to measure the protein levels of nephrin, podocin, desmin, NLRP3, pro-caspase1, cleaved caspase-1, ASC, NF-κB, and p-NF-κB. *** *p* < 0.001 vs. controls. **g**, The levels of IL-18 and IL-1β in cell culture supernatants were assessed by ELISA. *** *p* < 0.001 vs. controls. Data are presented as mean ± SEM from three independent experiments

The addition of adiponectin at various concentrations (1, 2, 4, 8 µg/ml) inhibited PA-induced cell death (Fig. 3 a) and apoptosis (Fig. 3b) in podocytes. The adiponectin treatment also inhibited ROS production in podocytes (Fig. 3 c). Considering the effectiveness and cost, we chose a dose of 2 µg/ml for the following adiponectin treatment. The results showed that adiponectin effectively increased the levels of podocyte marker proteins, including nephrin and podocyte, and decreased the expressions of desmin in MPC5 cells. The addition of adiponectin also inhibited the expressions of NLRP3 inflammasome-associated factors, including NLRP3, ASC, caspase-1, the phosphorylation of NF-κB in podocytes (Fig. 3d and e f). The concentrations of IL-18 and IL-1β in cells administered with both PA and adiponectin were also significantly lower compared to cells treated with PA alone (Fig. 3g). These results indicated that adiponectin ameliorated PA-induced cell injury and intracellular inflammation in podocytes.

### The knockdown of adiponectin gene induced intracellular inflammation, oxidative stress, and podocyte injury in MPC5 cells

**Fig. 4:**
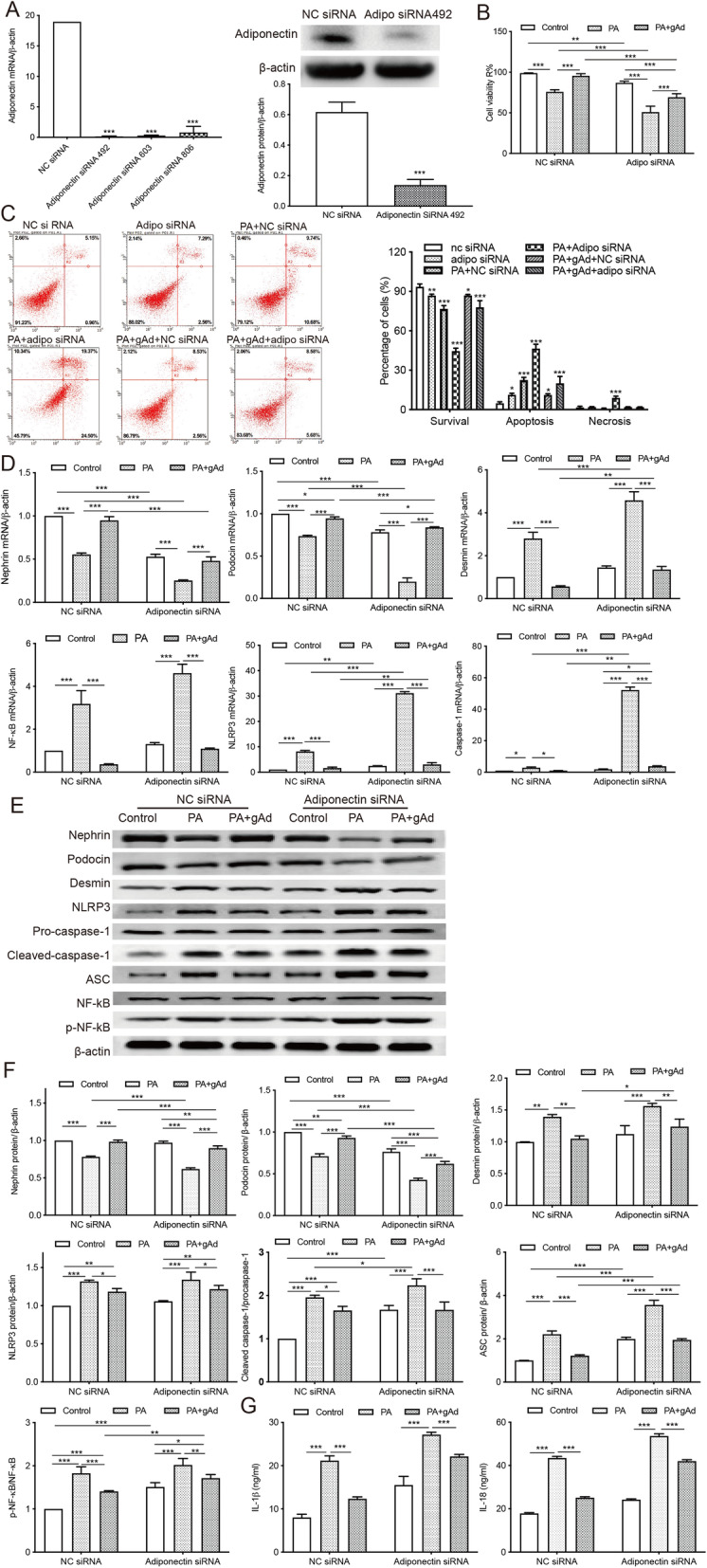
The knockdown of adiponectin exacerbates intracellular inflammation and apoptosis in MPC5 cells. Podocytes were transfected with siRNA against adiponectin or NC siRNA. **a**, The transfection efficiency was evaluated by qRT-PCR and Western blot. *** *p* < 0.001 vs. the NC siRNA group. **b**, CCK-8 assay was used to evaluate cell viability after transfection. ** *p* < 0.01, *** *p* < 0.001 vs. controls. **c**, The percentage of apoptotic cells was determined after the transfection. * *p* < 0.05, ** *p* < 0.01,*** *p* < 0.001 vs. controls. **d**, The mRNA expressions of nephrin, podocin, desmin, NLRP3, caspase-1, and NF-κB were evaluated by qRT-PCR. * *p* < 0.05, ** *p* < 0.01, *** *p* < 0.001 vs. controls. **e**-**f**, Western Blot analysis of nephrin, podocin, desmin, NLRP3, caspase-1, and NF-κB. * *p* < 0.05, ** *p* < 0.01, *** *p* < 0.001 vs. controls. **g**, Levels of inflammatory cytokines IL-18 and IL-1β were assessed by ELISA. *** *p* < 0.001 vs. controls. Data are presented as mean ± SEM from three independent experiments

To ascertain the protective effect of adiponectin on PA-treated podocytes, we transfected MPC5 cells with siRNA against adiponectin. The transfection efficiency was evaluated by qRT-PCR and Western blot (Fig. 4 a). The knockdown of adiponectin significantly reduced the cell viability and increased the apoptosis of podocytes. These effects were exacerbated by PA treatment but attenuated by the addition of adiponectin (Fig. 4b c). Compared to NC siRNA-transfected cells, the group delivered with siRNA against adiponectin showed decreased mRNA levels of nephrin and podocin, and significantly increased expressions of desmin, NLRP3, caspase-1, and NF-κB (Fig. 4d). In comparison to the NC siRNA group, cells with insufficient adiponectin expression displayed significantly upregulated protein levels of desmin, NLRP3, cleaved caspase-1, and p-NF-κB, which could be reversed by supplementation with adiponectin (Fig. 4e f). The insufficient expression of adiponectin also significantly increased the contents of inflammatory cytokines IL-18 and IL-1β in podocytes. These effects were exacerbated by PA treatment and could be reversed by the addition of adiponectin (Fig. 4g).

### NLRP3 inflammasome inhibitor MCC950 attenuated podocyte injury and intracellular oxidative stress in MPC5 cells

**Fig. 5:**
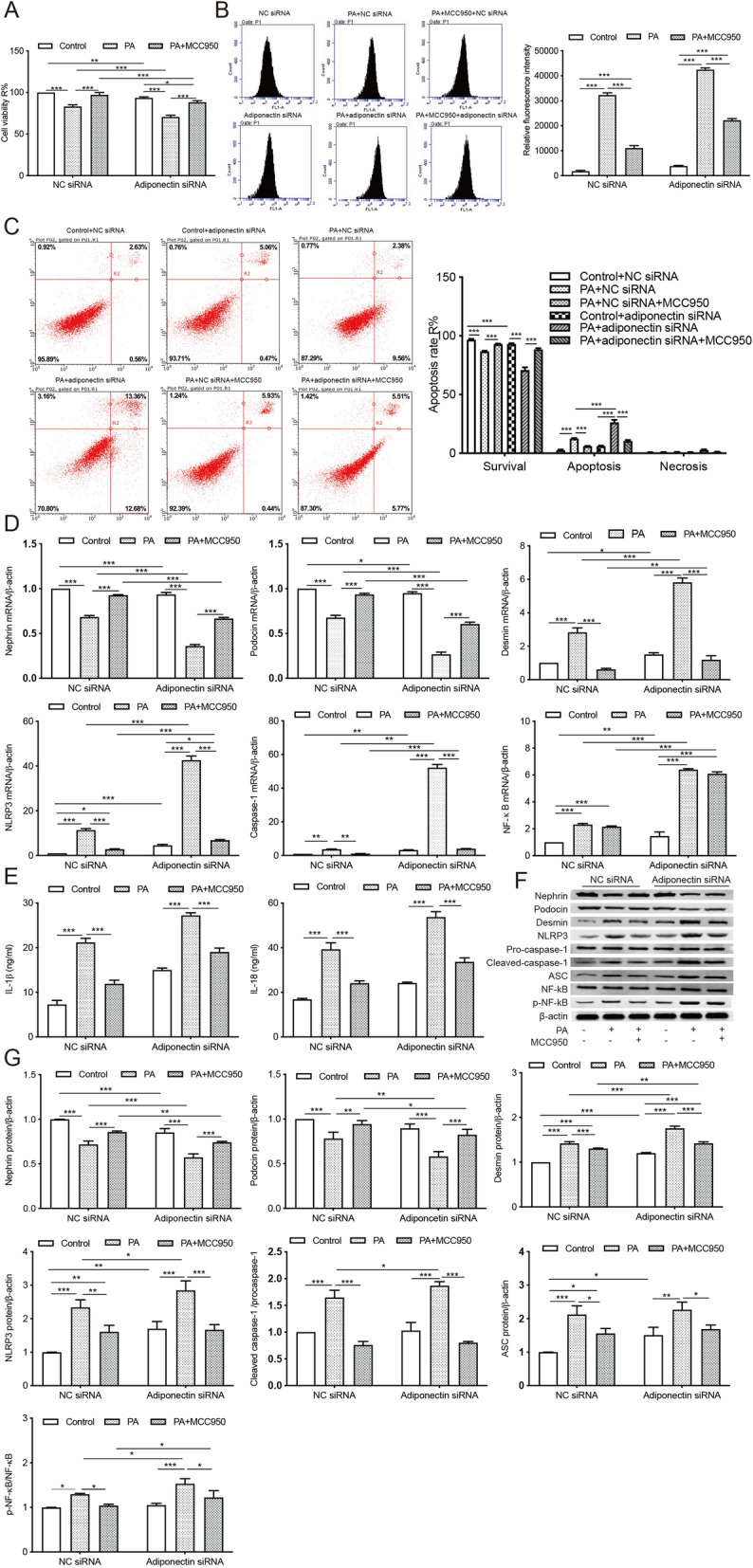
The NLRP3 inflammasome inhibitor MCC950 attenuated podocyte injury and intracellular oxidative stress induced by PA treatment or insufficient adiponectin expression. **a**, CCK-8 assay was performed to evaluate cell viability. * *p* < 0.05, *** *p* < 0.001 vs. controls. **b**, ROS production was assessed using a fluorometry assay. *** *p* < 0.001 vs. controls. **c**, The apoptosis was determined by flow cytometry. *** *p* < 0.001 vs. controls. **d**, qRT-PCR analysis of nephrin, podocin, desmin, NLRP3, caspase-1, and NF-κB. * *p* < 0.05, ** *p* < 0.01, *** *p* < 0.001 vs. controls. **e**, Levels of inflammatory cytokines IL-1β and IL-18 were measured by ELISA. *** *p* < 0.001 vs. controls. **f**-**g**, Western blot analysis determined the protein levels of nephrin, podocin, desmin,NLRP3, procaspase-1, cleaved caspase-1, ASC, p65, and phosphorylated p65. * *p* < 0.05, ** *p* < 0.01, *** *p* < 0.001 vs. controls. Data are presented as mean ± SEM from three independent experiments

Furthermore, podocytes transfected with siRNA against adiponectin or NC siRNA were treated with a potent NLRP3 inflammasome inhibitor MCC950 at 100 nM. MCC950 protected podocytes against PA- or si-Adiponectin-induced cell damage as shown by increased cell viability (Fig. 5 a), decreased ROS production (Fig. 5b), reduced apoptosis (Fig. 5 c), upregulated expressions of podocin and nephrin, and decreased levels of desmin, NLRP3, caspase-1, and ASC, IL-1β, IL-18, no matter whether the adiponectin-encoding gene was silenced or not (Fig. 5 c, d, e, f, g). However, the expression of NF-κB was not effected by MCC950 treatment. Taken together, we hypothesized that adiponectin protects against PA-induced podocyte injury by inhibiting cellular inflammation, apoptosis, and oxidative stress.

### PDTC prevented adiponectin from ameliorating PA-induced podocyte injury and suppressed NLRP3 activation

**Fig. 6:**
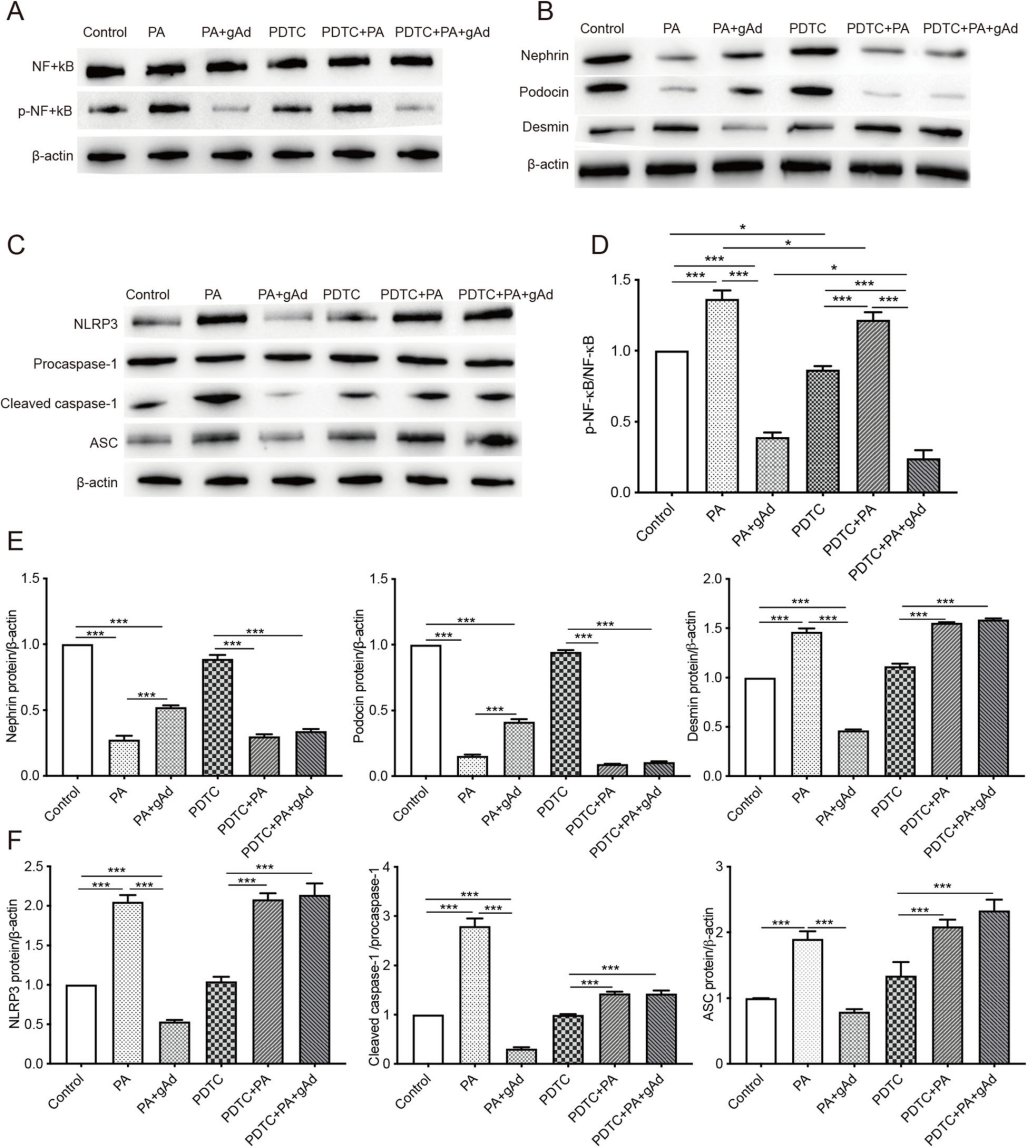
Effects of PDTC on NLRP3 inflammasome activation and expression of podocyte makers in PA-stimulated podocytes. Podocytes subjected to adiponectin treatment (0 or 2 µg/mL) were preconditioned with or without 50 µM PDTC for 2 h, followed by exposure to 150 µM PA for 24 h. **a**, **d** The levels of NF-κB and p-NF-κB were detected by Western blot. **b**, **e** The levels of podocyte makers (nephrin, podocin, and desmin) were determined by Western blot. **c**, **f** The levels of NLRP3 inflammasome (NLRP3, cleaved caspase-1, procaspase-1, and ASC) were measured by Western blot. * *p* < 0.05, *** *p* < 0.001 vs. the control group. Data are presented as mean ± SEM from three independent experiments

PDTC, an inhibitor of NF-κB, was used to explore the mechanism of action of adiponectin in PA-stimulated podocytes. As shown in Fig. 6 a and d, PDTC at 50 µM downregulated the expression of p-NF-κB in podocytes in the presence of PA (150 µM). The levels of nephrin and podocin were increased, while the expression of desmin was decreased due to the addition of adiponectin in PA-stimulated podocytes (Fig. 6b and e). Additionally, adiponectin exerted a significant inhibitory effect in NLRP3 inflammasome activation in PA-stimulated podocytes (Fig. 6 c, f). The simultaneous treatment of podocytes with PDTC (50 µM) and adiponectin (2 µg/mL) showed that PDTC blocked the effects of adiponectin in PA-stimulated podocytes (Fig. 6b c, e,  f). These results confirmed that adiponectin ameliorated podocyte injury and suppressed NLRP3 inflammasome activation in PA-stimulated podocytes by inhibiting the NF-κB pathway.

### Adiponectin attenuated HFD-induced renal injury in obese mice

**Fig. 7:**
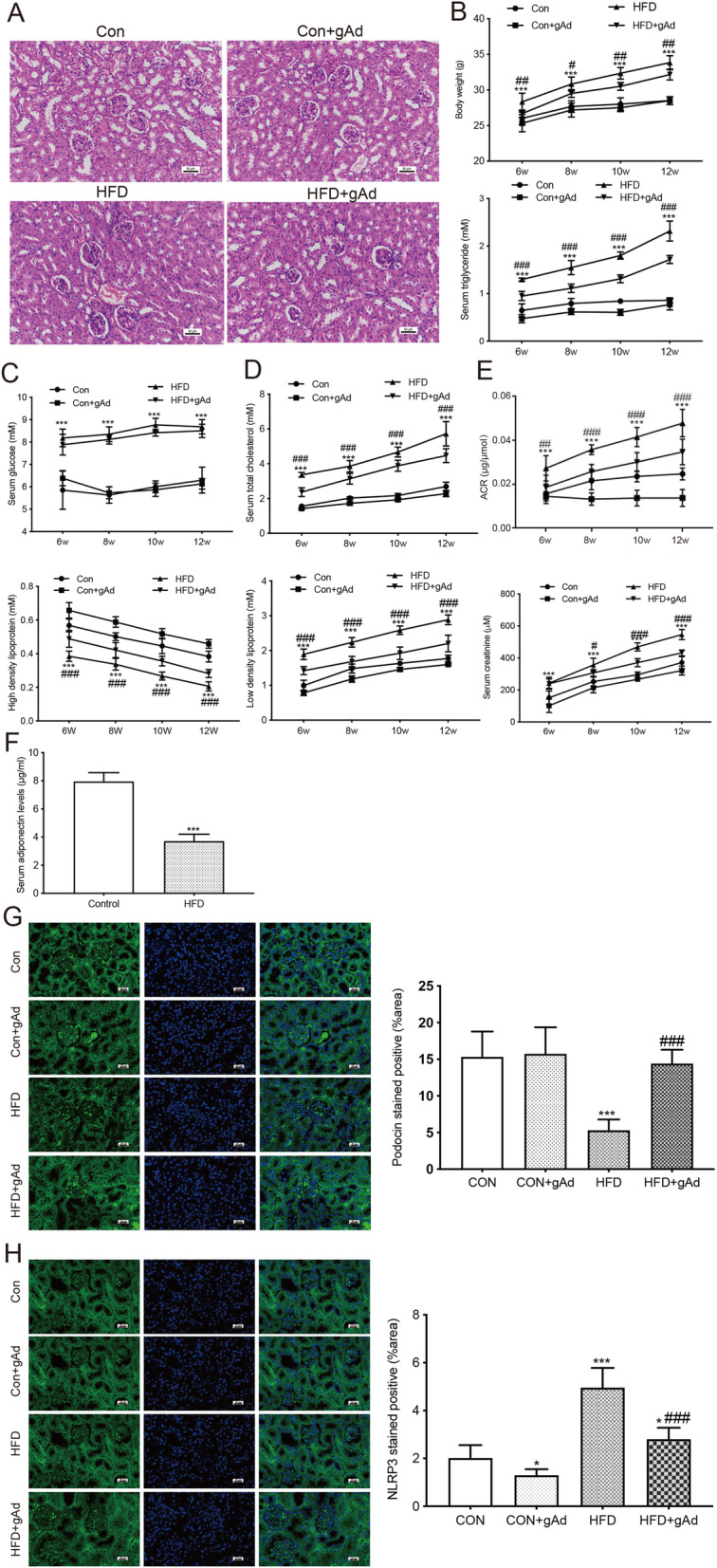
Adiponectin alleviated ORG in obese mice. **a**, Kidney tissue sections show podocytes in both groups. **b**-**c**, The body weight and blood glucose level in all animals were measured at 6, 8, 10, and 12 weeks of age). *** *p* < 0.001 vs. CON group, ^#^*p* < 0.05, ^##^*p* < 0.01 vs. HFD + gAd group. **d**, The serum concentrations of high-density lipoprotein, triglyceride, total cholesterol, and low-density lipoprotein were detected at 6, 8, 10, and 12 weeks of age. *** *p* < 0.001 vs. CON group, ^###^*p* < 0.001 vs. HFD + gAd group. **e** The levels of urinary ACR and serum were assessed at various time points (6, 8, 10, and 12 weeks of age). *** *p* < 0.001 vs. CON group, ^###^*p* < 0.001 vs. HFD + gAd group. **f** The level of serum adiponectin in mice was detected at 6 and 12 weeks of age. *** *p* < 0.001 vs. CON group. **g**-**h** Immunofluorescence stains of renal sections for podocin and NLRP3 at 12 weeks of age. * *p* < 0.05, *** *p* < 0.001 vs. CON group, ^###^*p* < 0.001 vs. HFD + gAd group. Data are presented as the mean ± SEM from three independent experiments

The size of podocytes in HFD-fed mice was significantly larger than that in the controls (Fig. 7 a), indicating that the mouse obesity model was successful established. The HFD group showed significantly increased body weight (Fig. 7b) and elevated levels of fasting blood glucose (Fig. 7 c), low-density lipoprotein cholesterol, triglyceride, total cholesterol (Fig. 7d), serum creatinine, and ACR (Fig. 7e) in comparison to the control group. The concentration of high-density lipoprotein cholesterol in the HFD group was significantly lower compared with the control group. The injection of adiponectin *via* tail vein reversed HFD-induced alterations in the above parameters, with the exception of blood glucose (Fig. 7b-e). The level of serum adiponectin in HFD-fed mice was lower than that in control animals (Fig. 7 f). The protection of adiponectin in HFD-induced kidney injury was also confirmed by increased podocin-positive area and lower number of NLRP3-positive cells in the HFD + gAd group versus the HFD group (Fig. 7g-h).

## Discussion

The incidence of ORG is expected to rise with the increasing prevalence of obesity and associated metabolic complications [25]. Valenci et al. has reported that urine albumin excretion rate in obese patients (average body mass index = 34.7 ± 5.7) was markedly elevated compared to the controls [26]. The elevated circulating level of FFAs is an essential hallmark of obesity, IR, and type 2 diabetes [27]. Podocytes are a key component of the glomerular filtration barrier (GFB) that prevents the filtration of plasma albumin. Nephrin and podocin are two podocyte marker proteins. In this study, we found that PA induced podocyte damage in a dose-dependent manner, as shown by downregulated expressions of nephrin and podocin, and impaired slit diaphragm, which led to the onset of proteinuria and subsequent deterioration. The expression of desmin has been shown to increase during proteinuria [28], making it a potential indicator of glomerular podocyte lesion. Here, we showed that the exposure to PA significantly upregulated the intracellular expression of desmin in podocytes at both mRNA and protein levels.

The activation of NLRP3 inflammasome by over-nutrition is involved in chronic sterile inflammation, which is a critical immune process in obesity [29]. Prior studies have shown that increased intracellular palmitate level induces the accumulation of an NLRP3 inflammasome activator, ceramide, and provides a continuous supply of DAMPs for the activation of the inflammasome [30]. Upon activation by DAMPs, NLRP3 interacts with ASC, whose caspase recruitment domain then binds to procaspase-1, forming the NLRP3 inflammasome and proteolytically activating IL-18 and IL-1β. Subsequently, the recruited procaspase-1 self-cleaves into the active caspase-1, inducing the secretion of IL-1β and IL-18 [31]. The involvement of NLRP3 inflammasome in obesity pathogenesis is supported by the results demonstrating that *As*c^−/−^ and *Nlrp3*^−/−^ mice were protected against HFD-induced IR and obesity [32]. The purinergic receptor P2 Χ 7, cathepsin B, and ROS-related signaling pathways have been proposed to contribute to NLRP3 inflammasome activation [33]. ROS regulates cell growth, differentiation, and migration, as well as the production of iNOS via redox-dependent NF-κB or mitogen-activated protein kinases signaling [34, 35]. It has been reported that the activation of NLRP3 can be blocked by ROS scavengers [36]. Consistent with previous results, our data showed PA-induced NLRP3 inflammasome activation, ROS production, and the release of IL-18 and IL-1β in podocytes, thereby accelerating the progression of renal injury (Fig. 2).

The crosstalk between NLRP3 inflammasome and ROS may be associated with ROS-induced NF-κB activation and maturation of IL-1β and caspase-1 [37, 38]. NF-κB signaling upregulates IL-1β precursor and *NLRP3* gene at the priming step of inflammasome activation [39]. Dysregulated activation of the NF-κB pathway is associated with progression of chronic kidney diseases, podocyte dysfunction, and aberrant nephrin expression [40–42]. In our study, adiponectin ameliorated PA-induced podocyte damage and inhibited NLRP3 inflammation activation in podocytes. The elevated expressions of p-p65 in PA-treated cells were also reduced by administration of adiponectin, which was consistent with previous findings. Meanwhile, the addition of adiponectin also significantly suppressed the secretion of IL-18 and IL-1β in PA-treated podocytes (Fig. 3).

Adiponectin is a protective adipocytokine circulating in three forms: high-order structures of oligomers, middle-molecular-weight hexamers, and low-molecular-weight trimers [43]. AdipoR1 is a functional adiponectin receptor expressed by glomerular cells and plays an essential role in mediating oxidative stress and cell survival [44]. The adiponectin dimers and monomers can be detected in urine samples from healthy individuals as they are small enough to cross GFB [45]. However, the high-molecular-weight adiponectin is only found in the urine of proteinuric and albuminuric patients, possibly resulting from the leakage through a dysfunctional GFB [46]. Rutkowski et al. established a mouse model of podocyte apoptosis (POD-ATTAC) and found that POD-ATTAC mice overexpressing adiponectin exhibited less interstitial fibrosis and recovered more rapidly, while the ones with insufficient adiponectin developed irreversible renal failure and albuminuria [25]. Also, prior work has shown that elevated urine hydrogen peroxide level in adiponectin-knockout mice was significantly reduced by supplementation with adiponectin [47]. To elucidate the protective mechanism of adiponectin on ORG, we silenced the gene encoding adiponectin in MPC5 cells. Our data revealed that the knockdown of adiponectin aggravated podocyte injury in cells treated with or without PA by inducing apoptotic cell death, secretion of inflammatory cytokines, and NLRP3 inflammasome activation, whereas the treatment of podocytes with adiponectin reversed these effects (Fig. 4).

MCC950 (or CRID3) is a highly specific NLRP3 inhibitor that may have therapeutic potential for the treatment of inflammation-related diseases [48]. Coll et al. demonstrated that MCC950 at nanomolar concentrations inhibited NLRP3 activation both in mice and in human cell lines [49]. The inhibitory effect of MCC950 on NLRP3 inflammasome in dendritic cells, myoblasts, macrophages, and microglia is also well described [50–53]. In our study, MCC950 protected podocytes against apoptosis cell death (Fig. 5 a), ROS production (Fig. 5b), cell apoptosis (Fig. 5 c), secretion of inflammatory cytokines IL-1β/IL-18 (Fig. 5e), and podocyte injury by inhibiting NLRP3 inflammation activation (Fig. 5d, f, g), while the activation of NF-κB signaling was not reversed by MCC950 treatment (Fig. 5d, f, g). NF-κB exists in the cytoplasm as an inactive form and is translocated into the nucleus after being activated [40]. Our data suggested that MCC950 treatment did not prevent the activation of NF-κB by pattern recognition receptors and tumor necrosis family receptors in response to various pro-inflammatory signals though inhibiting NLRP3 inflammation activation (Fig. 5d, f, g).

Previous studies have shown that the NLRP3 pathway is a downstream signaling pathway of NF-κB [54]. The canonical NF-κB pathway involves heterodimers of RelA/p65 and p105/p50, while the non-canonical pathway involves heterodimers of RelB and p100/p52. The canonical pathway is activated by pattern recognition receptors and tumor necrosis factor receptors in response to multiple pro-inflammatory signals, including cytokines. Upon stimulation, the IκB kinase complex phosphorylates IκB, the cytoplasmic repressor for the p50/p65 heterodimer, followed by IκB degradation and p65/p50 heterodimer release, and ultimately the translocation to the nucleus. Upon nuclear translocation, NF-κB transcription factors bind target genes and recruit chromatin modification enzymes and transcriptional cofactors to regulate gene expression [55].

PDTC, an inhibitor of NF-κB, was used to explore the mechanism of how adiponectin protected PA-stimulated podocytes. As shown in Fig. 6 a and d, PDTC treatment decreased PA-induced upregulation of p-NF-κB. The expressions of nephrin, podocin, and desmin in PDTC-treated cells were not affected by the addition of adiponectin (Fig. 6b and e). Furthermore, adiponectin did not lead to a significant decrease in NLRP3, cleaved caspase-1, and ASC in PDTC-treated podocytes subjected to PA stimulation (Fig. 6 c, f). These results confirmed that adiponectin ameliorated FFA-induced podocyte injury and NLRP3 inflammasome activation by inhibiting the NF-κB/NLRP3 pathway.

ORG encompasses a variety of pathological disorders, including focal segmental glomerulosclerosis, microalbuminuria, mesangial expansion, overt proteinuria, and glomerulomegaly. Obesity is typically accompanied by abnormal lipid metabolism. FFAs act with the low-density lipoprotein receptors on the surface of mesangial cells, inducing to the release of chemokines from macrophages, increasing the production of extracellular matrix and inflammatory factors, and ultimately leading to the increase in glomerular filtration rate and the development of glomerulosclerosis [56]. Here, we showed that the size of renal glomerulus, body weight, the levels of blood glucose, serum lipids except HDL levels, urinary ACR, and serum creatinine were significantly increased in HFD-fed group as compared to the controls, whereas adiponectin treatment ameliorated HFD-induced obesity and ORG in mice (Fig. 7 a-e). Additionally, there was no significant difference in the size of renal glomerulus, body weight, the levels of blood glucose, serum lipids (except HDL), urinary ACR, or serum creatinine between the CON and CON + gAd groups (Fig. 7 a-e). The HFD-fed group showed significantly decreased serum adiponectin level (Fig. 7 f) and podocin expression, and increased NLRP3-stained areas as compared to the controls (Fig. 7g-h), whereas adiponectin treatment attenuated the effects of HFD on the expression of these proteins (Fig. 7g-h).

There are some limitations in the present study. First, the lipid content, podocyte apoptosis rate, the expression level of marker proteins and inflammatory factors were not confirmed *in vivo*. Further investigations on apoptosis, inflammation-related proteins and cytokines, and the activation of oxidative stress markers are needed to better characterize the ORG model. The renal expressions of AdipoR1 and AdipoR2 could also be detected in HFD-fed mice to explore the involvement of the RAAS pathway in ORG.

## Conclusions

In conclusion, this study shows that adiponectin ameliorated FFA-induced podocyte injury *in vitro* via downregulating the ROS/NF-κB/NLRP3 pathway. Adiponectin treatment ameliorated HFD-induced obesity and ORG in mice. These findings support the potential therapeutic use of adiponectin in ORG.

## Supplementary Information

**Additional file 1:**

## Data Availability

All data generated or analyzed during this study are included in this published article.

## Abbreviations

ACR: Urinary protein excretion/urinary creatinine
AdipoR1: Adiponectin receptor1
AdipoR2: Adiponectin receptor 2
ASC: Apoptosis-associated speck-like protein
BODIPY: Boron-dipyrromethene
CON: Control
DAMPs: Damage-associated molecular patterns
DMEM: Dulbecco’s Modified Eagle's Medium
FBS: Fetal bovine serum
FFA: Free fatty acids
gAd: Globular adiponectin
GFB: Glomerular filtration barrier
HFD: High fat diet
IR: Insulin resistance
MPC5: Murine podocyte clone 5
NC: Negative control
NF-κB: Nuclear factor-κB
NLRP3: Recombinant NLR family, pyrin domain containing protein 3
ORG: Obesity-related glomerulopathy
PA: Palmitic acid
PDTC: Pyrrolidine dithiocarbamate
qRT-PCR: Quantitative real-time polymerase chain reaction
ROS: Reactive oxygen species
siRNA: Small-interfering RNA

## References

[1] Weiwei C, Runlin G, Lisheng L, Manlu Z, Wen W, Yongjun W (2016). Outline of the report on cardiovascular diseases in China, 2014. Eur Heart J Suppl.

[2] Pazos F (2020). Range of adiposity and cardiorenal syndrome. World J Diabetes.

[3] Tsuboi N, Koike K, Hirano K, Utsunomiya Y, Kawamura T, Hosoya T (2013). Clinical features and long-term renal outcomes of Japanese patients with obesity-related glomerulopathy. Clin Exp Nephrol.

[4] Xu T, Sheng Z, Yao L (2017). Obesity-related glomerulopathy: pathogenesis, pathologic, clinical characteristics and treatment. Front Med.

[5] D’Agati VD, Chagnac A, de Vries AP, Levi M, Porrini E, Herman-Edelstein M (2016). Obesity-related glomerulopathy: clinical and pathologic characteristics and pathogenesis. Nat Rev Nephrol.

[6] Chen HM, Li SJ, Chen HP, Wang QW, Li LS, Liu ZH (2008). Obesity-related glomerulopathy in China: a case series of 90 patients. Am J Kidney Dis.

[7] Akoumianakis I, Antoniades C (2017). The interplay between adipose tissue and the cardiovascular system: is fat always bad?. Cardiovasc Res.

[8] Rüster C, Wolf G (2013). The role of the renin-angiotensin-aldosterone system in obesity-related renal diseases. Semin Nephrol.

[9] Manabe I (2011). Chronic inflammation links cardiovascular, metabolic and renal diseases. Circ J.

[10] Fogo AB (2015). Causes and pathogenesis of focal segmental glomerulosclerosis. Nat Rev Nephrol.

[11] Ni Y, Wang X, Yin X, Li Y, Liu X, Wang H (2018). Plectin protects podocytes from adriamycin-induced apoptosis and F-actin cytoskeletal disruption through the integrin α6β4/FAK/p38 MAPK pathway. J Cell Mol Med.

[12] Tagawa A, Yasuda M, Kume S, Yamahara K, Nakazawa J, Chin-Kanasaki M (2016). Impaired Podocyte Autophagy Exacerbates Proteinuria in Diabetic Nephropathy. Diabetes.

[13] Greka A, Mundel P (2012). Cell biology and pathology of podocytes. Annu Rev Physiol.

[14] Takemura Y, Ouchi N, Shibata R, Aprahamian T, Kirber MT, Summer RS (2007). Adiponectin modulates inflammatory reactions via calreticulin receptor-dependent clearance of early apoptotic bodies. J Clin Invest.

[15] Ruan H, Dong LQ (2016). Adiponectin signaling and function in insulin target tissues. J Mol Cell Biol.

[16] Achari AE, Jain SK. Adiponectin, a Therapeutic Target for Obesity, Diabetes, and Endothelial Dysfunction. Int J Mol Sci. 2017;18. 10.3390/ijms18061321 PMC5486142 28635626

[17] Kita S, Fukuda S, Maeda N, Shimomura I. Native adiponectin in serum binds to mammalian cells expressing T-cadherin, but not AdipoRs or calreticulin. Elife. 2019;8. 10.7554/eLife.48675 PMC6822988 31647413

[18] Clark JL, Taylor CG, Zahradka P (2017). Exploring the Cardio-metabolic Relevance of T-cadherin: A Pleiotropic Adiponectin Receptor. Endocr Metab Immune Disord Drug Targets.

[19] Yamauchi T, Kadowaki T (2008). Physiological and pathophysiological roles of adiponectin and adiponectin receptors in the integrated regulation of metabolic and cardiovascular diseases. Int J Obes (Lond).

[20] Ohashi K, Iwatani H, Kihara S, Nakagawa Y, Komura N, Fujita K (2007). Exacerbation of albuminuria and renal fibrosis in subtotal renal ablation model of adiponectin-knockout mice. Arterioscler Thromb Vasc Biol.

[21] Lee HM, Kim JJ, Kim HJ, Shong M, Ku BJ, Jo EK (2013). Upregulated NLRP3 inflammasome activation in patients with type 2 diabetes. Diabetes.

[22] Legrand-Poels S, Esser N, L’Homme L, Scheen A, Paquot N, Piette J (2014). Free fatty acids as modulators of the NLRP3 inflammasome in obesity/type 2 diabetes. Biochem Pharmacol.

[23] Stienstra R, Tack CJ, Kanneganti TD, Joosten LA, Netea MG (2012). The inflammasome puts obesity in the danger zone. Cell Metab.

[24] Tian D, Qiu Y, Zhan Y, Li X, Zhi X, Wang X (2012). Overexpression of steroidogenic acute regulatory protein in rat aortic endothelial cells attenuates palmitic acid-induced inflammation and reduction in nitric oxide bioavailability. Cardiovasc Diabetol.

[25] Sweiss N, Sharma K (2014). Adiponectin effects on the kidney. Best Pract Res Clin Endocrinol Metab.

[26] Barnes VA, Treiber FA, Davis H, Kelley TR, Strong WB (1998). Central adiposity and hemodynamic functioning at rest and during stress in adolescents. Int J Obes Relat Metab Disord.

[27] Krebs M, Roden M (2005). Molecular mechanisms of lipid-induced insulin resistance in muscle, liver and vasculature. Diabetes Obes Metab.

[28] Yaoita E, Franke WW, Yamamoto T, Kawasaki K, Kihara I (1999). Identification of renal podocytes in multiple species: higher vertebrates are vimentin positive/lower vertebrates are desmin positive. Histochem Cell Biol.

[29] Traba J, Sack MN (2017). The role of caloric load and mitochondrial homeostasis in the regulation of the NLRP3 inflammasome. Cell Mol Life Sci.

[30] Zhong Y, Kinio A, Saleh M (2013). Functions of NOD-Like Receptors in Human Diseases. Front Immunol.

[31] Shao BZ, Xu ZQ, Han BZ, Su DF, Liu C (2015). NLRP3 inflammasome and its inhibitors: a review. Front Pharmacol.

[32] Stienstra R, van Diepen JA, Tack CJ, Zaki MH, van de Veerdonk FL, Perera D (2011). Inflammasome is a central player in the induction of obesity and insulin resistance. Proc Natl Acad Sci U S A.

[33] Zhou R, Tardivel A, Thorens B, Choi I, Tschopp J (2010). Thioredoxin-interacting protein links oxidative stress to inflammasome activation. Nat Immunol.

[34] Martinon F (2010). Signaling by ROS drives inflammasome activation. Eur J Immunol.

[35] Ray PD, Huang BW, Tsuji Y (2012). Reactive oxygen species (ROS) homeostasis and redox regulation in cellular signaling. Cell Signal.

[36] Dostert C, Pétrilli V, Van Bruggen R, Steele C, Mossman BT, Tschopp J (2008). Innate immune activation through Nalp3 inflammasome sensing of asbestos and silica. Science.

[37] Liao PC, Chao LK, Chou JC, Dong WC, Lin CN, Lin CY (2013). Lipopolysaccharide/adenosine triphosphate-mediated signal transduction in the regulation of NLRP3 protein expression and caspase-1-mediated interleukin-1β secretion. Inflamm Res.

[38] Corsini E, Galbiati V, Nikitovic D, Tsatsakis AM (2013). Role of oxidative stress in chemical allergens induced skin cells activation. Food Chem Toxicol.

[39] Tvarijonaviciute A, Ceron JJ, Holden SL, Cuthbertson DJ, Biourge V, Morris PJ (2012). Obesity-related metabolic dysfunction in dogs: a comparison with human metabolic syndrome. BMC Vet Res.

[40] Brähler S, Ising C, Hagmann H, Rasmus M, Hoehne M, Kurschat C (2012). Intrinsic proinflammatory signaling in podocytes contributes to podocyte damage and prolonged proteinuria. Am J Physiol Renal Physiol.

[41] Yamashita M, Millward CA, Inoshita H, Saikia P, Chattopadhyay S, Sen GC (2013). Antiviral innate immunity disturbs podocyte cell function. J Innate Immun.

[42] Liu R, Zhong Y, Li X, Chen H, Jim B, Zhou MM (2014). Role of transcription factor acetylation in diabetic kidney disease. Diabetes.

[43] Esmaili S, Xu A, George J (2014). The multifaceted and controversial immunometabolic actions of adiponectin. Trends Endocrinol Metab.

[44] Cammisotto PG, Bendayan M (2008). Adiponectin stimulates phosphorylation of AMP-activated protein kinase alpha in renal glomeruli. J Mol Histol.

[45] Shimotomai T, Kakei M, Narita T, Koshimura J, Hosoba M, Kato M (2005). Enhanced urinary adiponectin excretion in IgA-nephropathy patients with proteinuria. Ren Fail.

[46] Shen YY, Hughes JT, Charlesworth JA, Kelly JJ, Peake PW (2008). Adiponectin is present in the urine in its native conformation, and specifically reduces the secretion of MCP-1 by proximal tubular cells. Nephrology (Carlton).

[47] Ebenezer PJ, Mariappan N, Elks CM, Haque M, Soltani Z, Reisin E (2009). Effects of pyrrolidine dithiocarbamate on high-fat diet-induced metabolic and renal alterations in rats. Life Sci.

[48] Coll RC, Robertson AA, Chae JJ, Higgins SC, Muñoz-Planillo R, Inserra MC (2015). A small-molecule inhibitor of the NLRP3 inflammasome for the treatment of inflammatory diseases. Nat Med.

[49] Fais RS, Rodrigues FL, Pereira CA, Mendes AC, Mestriner F, Tostes RC (2019). The inflammasome NLRP3 plays a dual role on mouse corpora cavernosa relaxation. Sci Rep.

[50] Primiano MJ, Lefker BA, Bowman MR, Bree AG, Hubeau C, Bonin PD (2016). Efficacy and Pharmacology of the NLRP3 Inflammasome Inhibitor CP-456,773 (CRID3) in Murine Models of Dermal and Pulmonary Inflammation. J Immunol.

[51] Nalbandian A, Khan AA, Srivastava R, Llewellyn KJ, Tan B, Shukr N (2017). Activation of the NLRP3 Inflammasome Is Associated with Valosin-Containing Protein Myopathy. Inflammation.

[52] Ludwig-Portugall I, Bartok E, Dhana E, Evers BD, Primiano MJ, Hall JP (2016). An NLRP3-specific inflammasome inhibitor attenuates crystal-induced kidney fibrosis in mice. Kidney Int..

[53] Dempsey C, Rubio Araiz A, Bryson KJ, Finucane O, Larkin C, Mills EL (2017). Inhibiting the NLRP3 inflammasome with MCC950 promotes non-phlogistic clearance of amyloid-β and cognitive function in APP/PS1 mice. Brain Behav Immun.

[54] Sutterwala FS, Haasken S, Cassel SL (2014). Mechanism of NLRP3 inflammasome activation. Ann N Y Acad Sci.

[55] Zhao X, Hsu KS, Lim JH, Bruggeman LA, Kao HY (2015). α-Actinin 4 potentiates nuclear factor κ-light-chain-enhancer of activated B-cell (NF-κB) activity in podocytes independent of its cytoplasmic actin binding function. J Biol Chem.

[56] Gai Z, Gui T, Hiller C, Kullak-Ublick GA (2016). Farnesoid X Receptor Protects against Kidney Injury in Uninephrectomized Obese Mice. J Biol Chem.

