# Supplementary material for: Adiponectin protects obesity-related glomerulopathy by inhibiting ROS/NF-κB/NLRP3 inflammation pathway
Source: BMC Nephrol. 2021 Jun 10;22:218. doi: 10.1186/s12882-021-02391-1 (PMC8191043; doi:10.1186/s12882-021-02391-1)
Supplement: Supplementary file 1 — Additional file 1: [file 12882_2021_2391_MOESM1_ESM.pptx]

## Slide 1
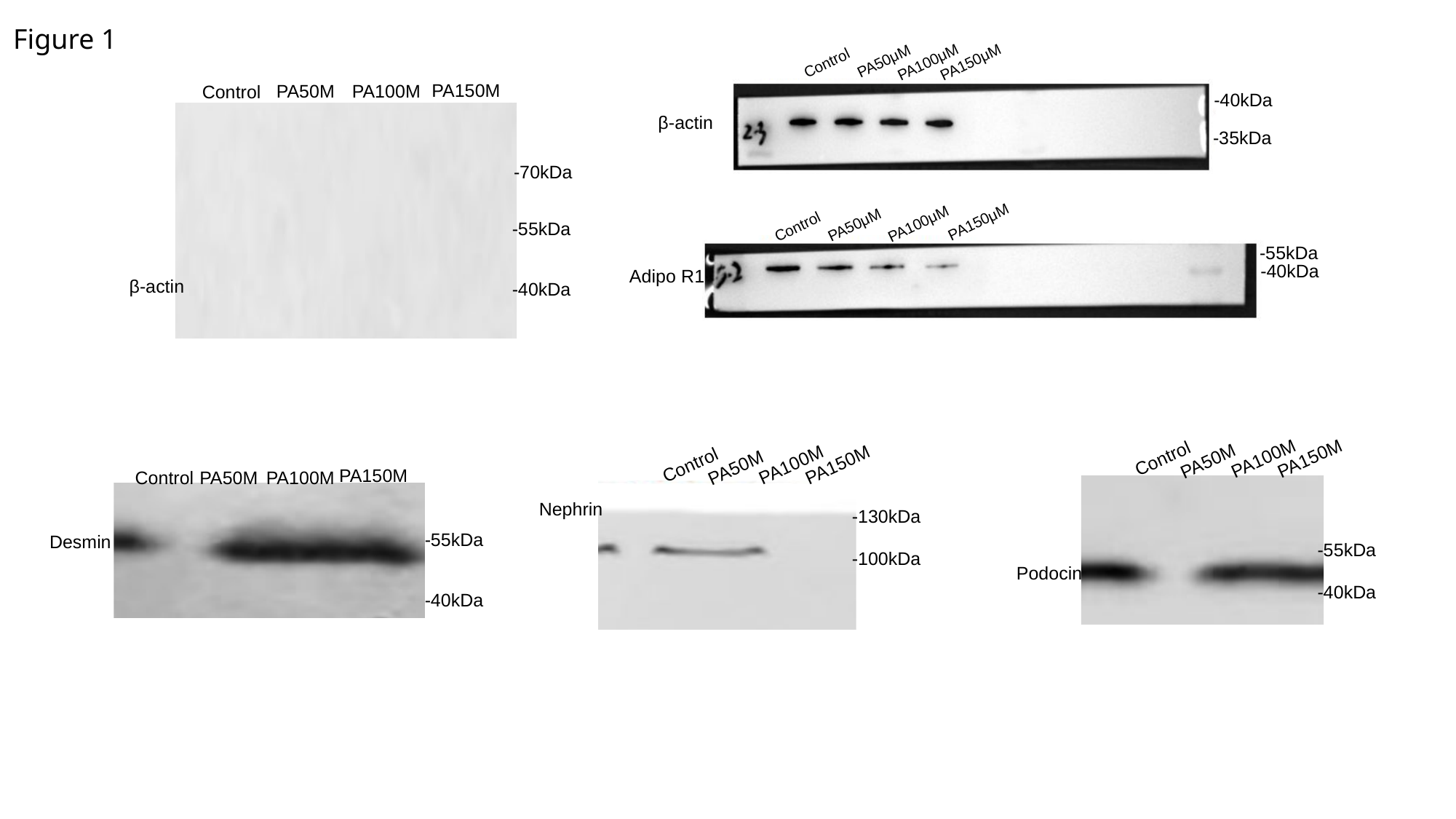

Figure 1
PA50μM
PA100μM
PA150μM
Control
-40kDa
β-actin
-35kDa
PA150M
PA100M
PA50M
Control
-70kDa
-55kDa
β-actin
-40kDa
PA150μM
PA100μM
PA50μM
Control
-55kDa
-40kDa
Adipo R1
Control
PA100M
PA150M
PA50M
-55kDa
Podocin
-40kDa
Control
PA100M
PA150M
PA50M
Nephrin
-130kDa
-100kDa
PA150M
Control
PA50M
PA100M
-55kDa
Desmin
-40kDa

## Slide 2
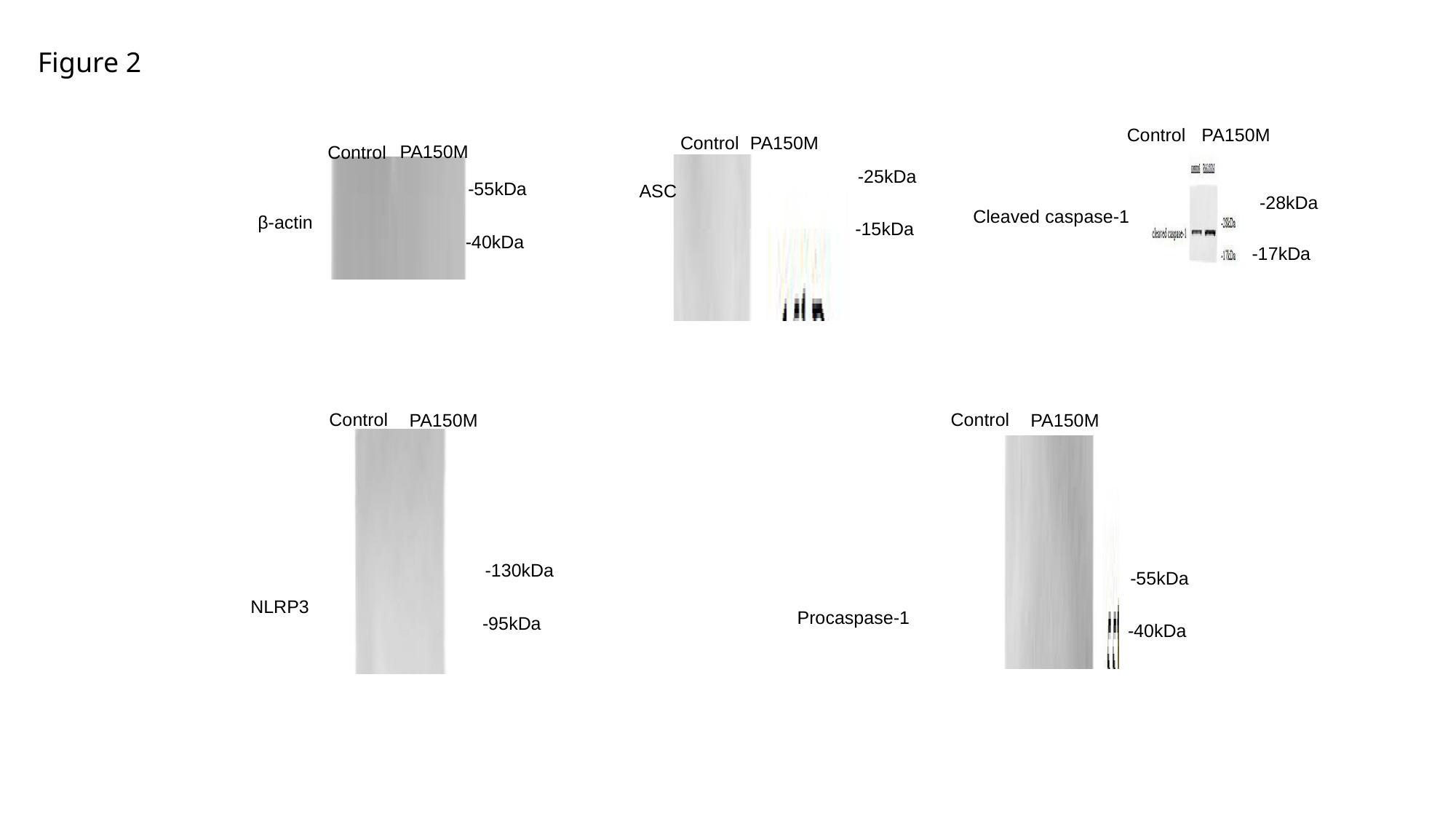

Figure 2
PA150M
Control
-28kDa
Cleaved caspase-1
-17kDa
PA150M
Control
-25kDa
ASC
-15kDa
PA150M
Control
-55kDa
β-actin
-40kDa
Control
PA150M
-130kDa
NLRP3
-95kDa
Control
PA150M
-55kDa
Procaspase-1
-40kDa

## Slide 3
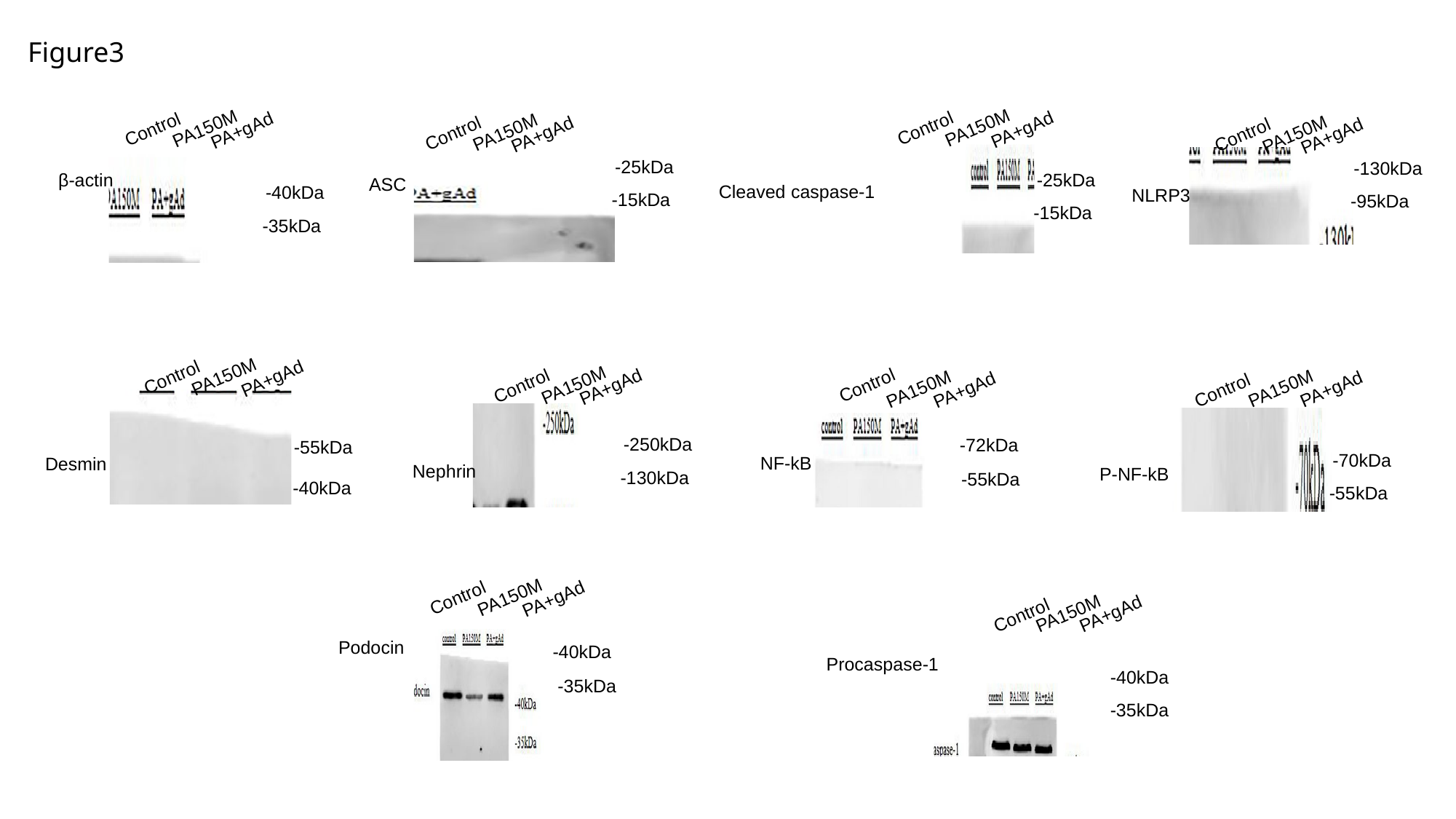

Figure3
PA150M
Control
PA+gAd
-25kDa
Cleaved caspase-1
-15kDa
PA150M
Control
PA+gAd
β-actin
-40kDa
-35kDa
PA150M
Control
PA+gAd
-25kDa
ASC
-15kDa
PA150M
Control
PA+gAd
-130kDa
NLRP3
-95kDa
PA150M
Control
PA+gAd
-55kDa
Desmin
-40kDa
PA150M
Control
PA+gAd
-250kDa
Nephrin
-130kDa
Control
PA150M
-72kDa
NF-kB
-55kDa
PA150M
Control
-70kDa
P-NF-kB
-55kDa
PA+gAd
PA+gAd
PA150M
Control
PA+gAd
Podocin
-40kDa
-35kDa
PA150M
PA+gAd
Control
Procaspase-1
-40kDa
-35kDa

## Slide 4
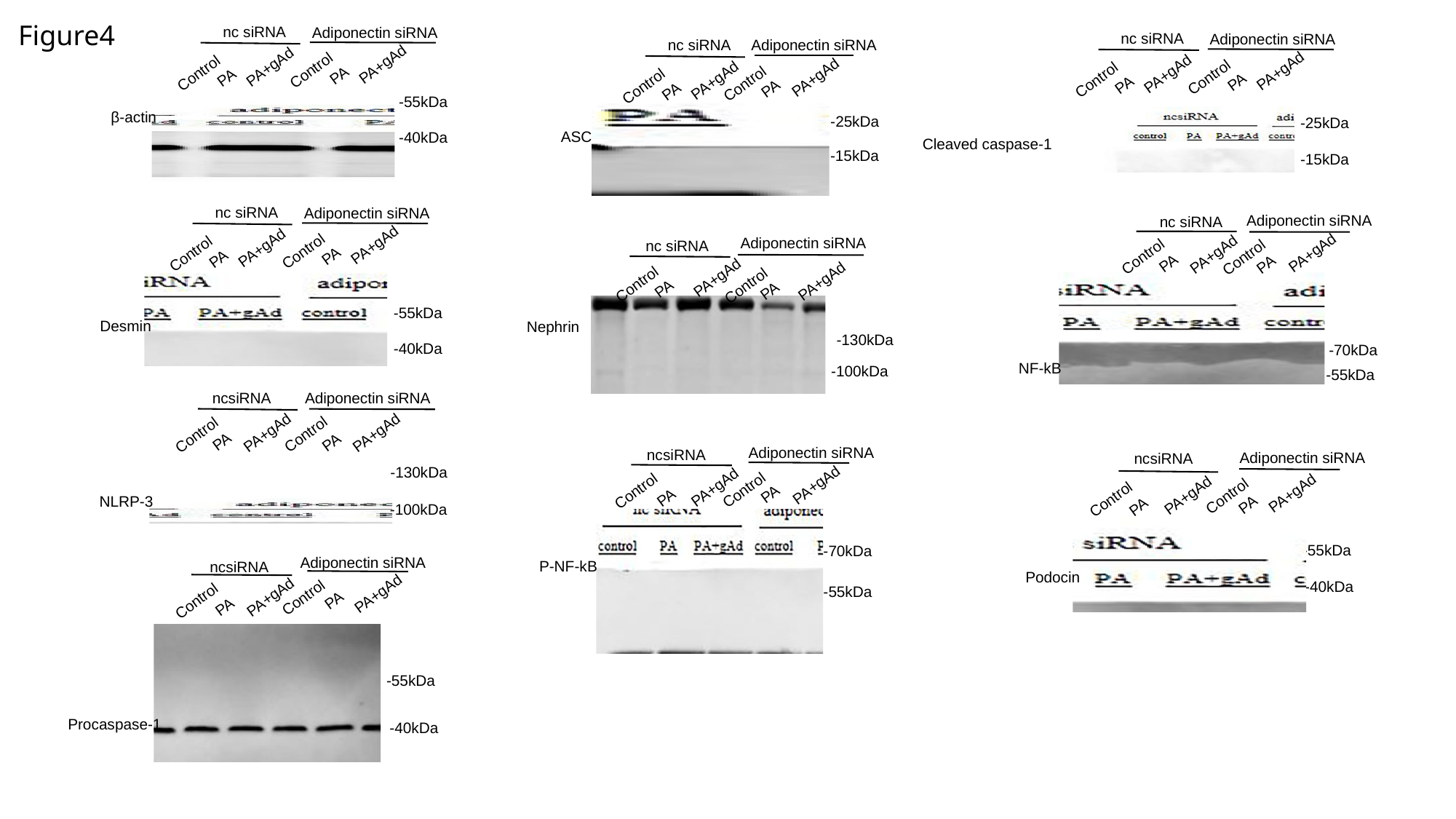

Figure4
nc siRNA
Adiponectin siRNA
PA+gAd
PA+gAd
Control
Control
PA
PA
-55kDa
β-actin
-40kDa
nc siRNA
Adiponectin siRNA
PA+gAd
PA+gAd
Control
Control
PA
PA
-25kDa
Cleaved caspase-1
-15kDa
nc siRNA
Adiponectin siRNA
PA+gAd
PA+gAd
Control
Control
PA
PA
-25kDa
ASC
-15kDa
nc siRNA
Adiponectin siRNA
PA+gAd
PA+gAd
Control
Control
PA
PA
-55kDa
Desmin
-40kDa
Adiponectin siRNA
nc siRNA
PA+gAd
PA+gAd
Control
Control
PA
PA
-70kDa
NF-kB
-55kDa
Adiponectin siRNA
nc siRNA
PA+gAd
PA+gAd
Control
Control
PA
PA
Nephrin
-130kDa
-100kDa
Adiponectin siRNA
ncsiRNA
PA+gAd
PA+gAd
Control
Control
PA
PA
-130kDa
NLRP-3
-100kDa
Adiponectin siRNA
ncsiRNA
PA+gAd
PA+gAd
Control
Control
PA
PA
-70kDa
P-NF-kB
-55kDa
Adiponectin siRNA
ncsiRNA
PA+gAd
PA+gAd
Control
Control
PA
PA
Podocin
-55kDa
Adiponectin siRNA
ncsiRNA
PA+gAd
PA+gAd
Control
PA
Control
PA
-55kDa
Procaspase-1
-40kDa
-40kDa

## Slide 5
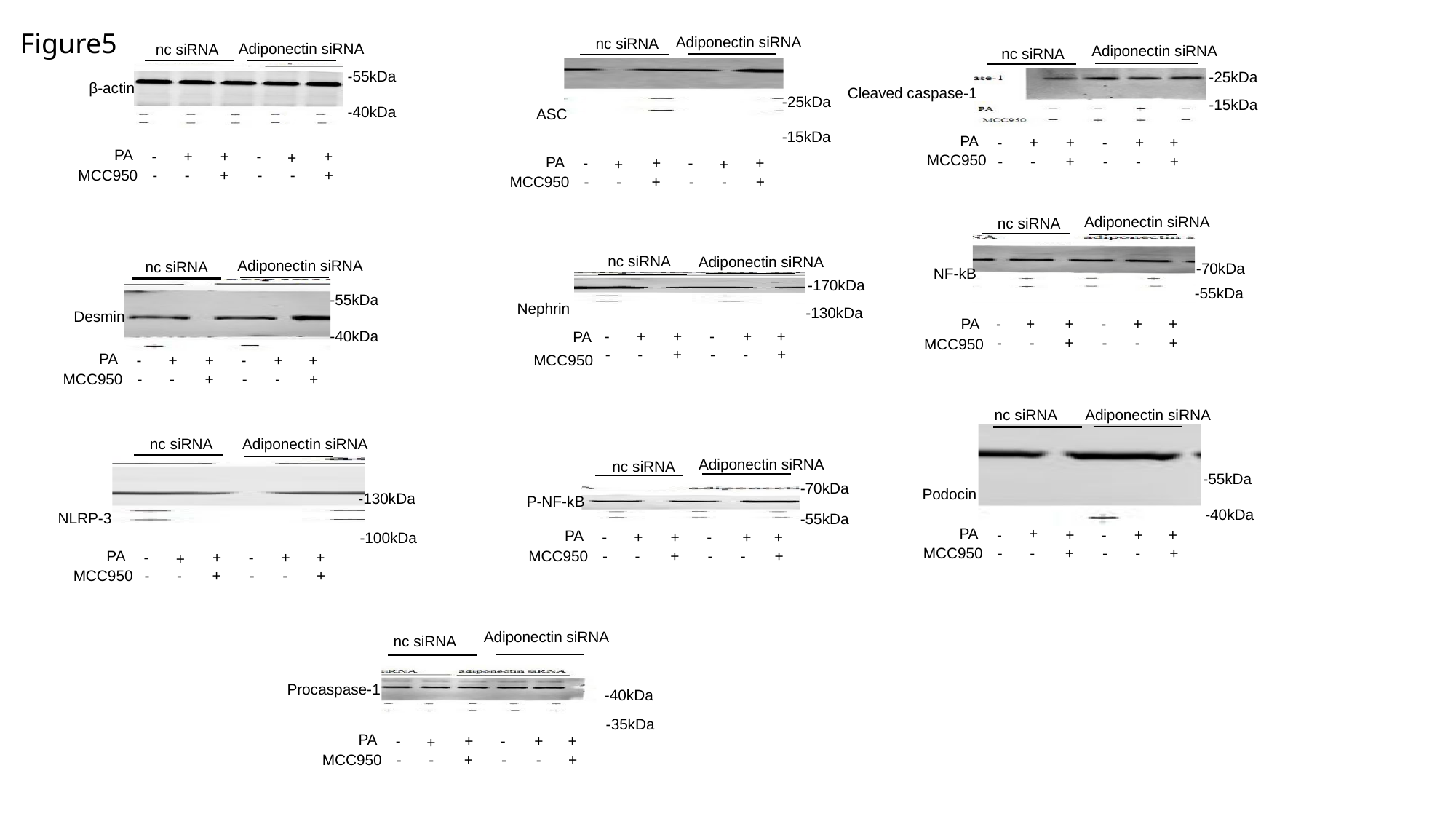

Figure5
Adiponectin siRNA
nc siRNA
-25kDa
ASC
-15kDa
PA
-
+
-
+
+
+
-
-
+
-
-
+
MCC950
Adiponectin siRNA
nc siRNA
Adiponectin siRNA
nc siRNA
-25kDa
Cleaved caspase-1
-15kDa
PA
-
+
+
-
+
+
-
-
+
-
-
+
MCC950
-55kDa
β-actin
-40kDa
PA
-
+
+
-
+
+
-
-
+
-
-
+
MCC950
Adiponectin siRNA
nc siRNA
-70kDa
NF-kB
-55kDa
PA
-
+
+
-
+
+
-
-
+
-
-
+
nc siRNA
Adiponectin siRNA
-170kDa
Nephrin
-130kDa
-
+
+
-
+
+
-
-
+
-
-
+
PA
MCC950
Adiponectin siRNA
nc siRNA
-55kDa
Desmin
-40kDa
PA
-
+
+
-
+
+
-
-
+
-
-
+
MCC950
MCC950
nc siRNA
Adiponectin siRNA
-55kDa
Podocin
-40kDa
PA
+
-
+
-
+
+
-
-
+
-
-
+
MCC950
Adiponectin siRNA
nc siRNA
-130kDa
NLRP-3
-100kDa
PA
-
+
-
+
+
+
-
-
+
-
-
+
MCC950
Adiponectin siRNA
nc siRNA
-70kDa
P-NF-kB
-55kDa
PA
-
+
+
-
+
+
-
-
+
-
-
+
MCC950
Adiponectin siRNA
nc siRNA
Procaspase-1
-40kDa
-35kDa
PA
-
+
-
+
+
+
-
-
+
-
-
+
MCC950

## Slide 6
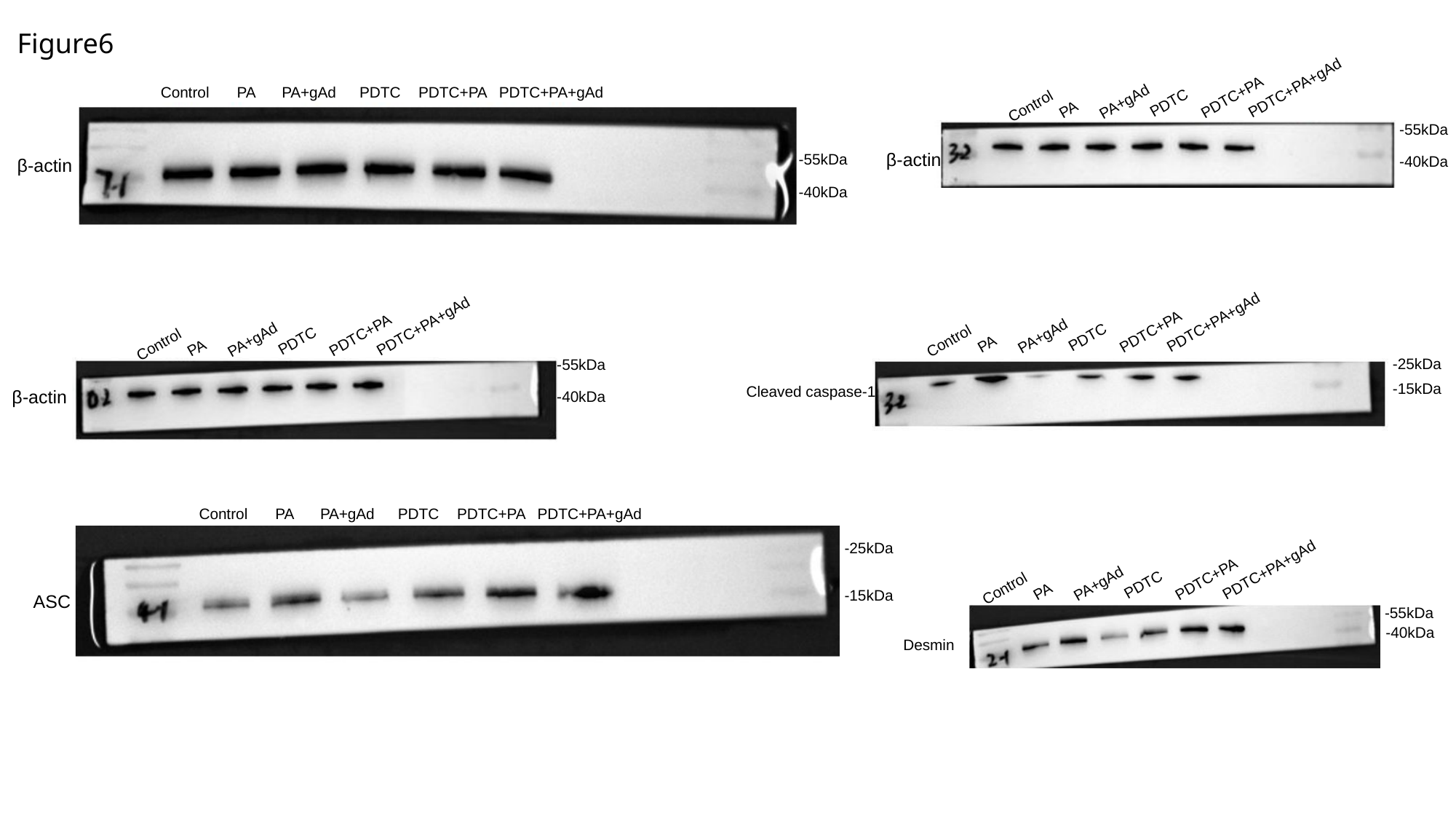

Figure6
PDTC+PA+gAd
Control
PA
PA+gAd
PDTC
PDTC+PA
PDTC+PA+gAd
PDTC+PA
PA+gAd
PDTC
Control
PA
-55kDa
β-actin
-55kDa
-40kDa
β-actin
-40kDa
PDTC+PA+gAd
PDTC+PA
PA+gAd
PDTC
Control
PA
-25kDa
-15kDa
Cleaved caspase-1
PDTC+PA+gAd
PDTC+PA
PA+gAd
PDTC
Control
PA
-55kDa
β-actin
-40kDa
Control
PA
PA+gAd
PDTC
PDTC+PA
PDTC+PA+gAd
-25kDa
-15kDa
ASC
PDTC+PA+gAd
PDTC+PA
PA+gAd
PDTC
Control
PA
-55kDa
-40kDa
Desmin

## Slide 7
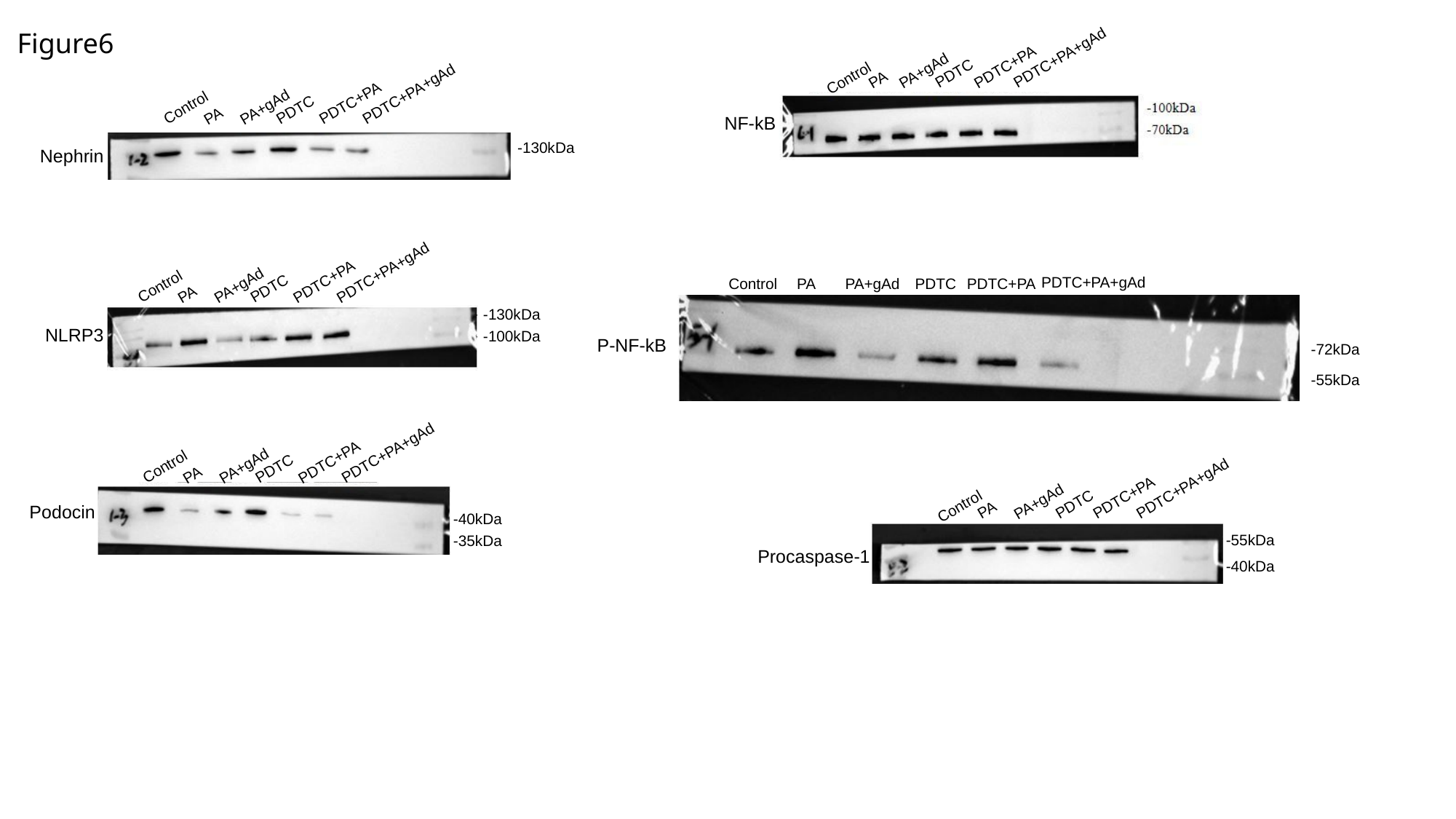

Figure6
PDTC+PA+gAd
PDTC+PA
PA+gAd
PDTC
Control
PA
PDTC+PA+gAd
PDTC+PA
PA+gAd
Control
PDTC
PA
NF-kB
-130kDa
Nephrin
PDTC+PA+gAd
PDTC+PA
PA+gAd
Control
PDTC
PA
-130kDa
NLRP3
-100kDa
PDTC+PA+gAd
PDTC
PA+gAd
PA
PDTC+PA
Control
P-NF-kB
-72kDa
-55kDa
PDTC+PA+gAd
PDTC+PA
PA+gAd
Control
PDTC
PA
Podocin
-40kDa
-35kDa
PDTC+PA+gAd
PDTC+PA
PA+gAd
PDTC
Control
PA
-55kDa
Procaspase-1
-40kDa
